# Identification of Habitat-Specific Biomes of Aquatic Fungal Communities Using a Comprehensive Nearly Full-Length 18S rRNA Dataset Enriched with Contextual Data

**DOI:** 10.1371/journal.pone.0134377

**Published:** 2015-07-30

**Authors:** Katrin Panzer, Pelin Yilmaz, Michael Weiß, Lothar Reich, Michael Richter, Jutta Wiese, Rolf Schmaljohann, Antje Labes, Johannes F. Imhoff, Frank Oliver Glöckner, Marlis Reich

**Affiliations:** 1 University of Bremen, Molecular Ecology, FB2, Leobener Str. 2, 28359, Bremen, Germany; 2 Jacobs University Bremen gGmbH, Department of Life Sciences and Chemistry, Campusring 1, 28759, Bremen, Germany; 3 Max Planck Institute for Marine Microbiology, Microbial Genomics and Bioinformatics Research Group, Celsiusstr.1, 28359, Bremen, Germany; 4 University of Tübingen, Department of Biology, Auf der Morgenstelle 1, 72076 Tübingen, Germany; 5 Potsdam Transfer, August-Bebel-Straße 89, Haus 7, 14482, Potsdam, Germany; 6 GEOMAR Helmholtz Centre for Ocean Research Kiel, Marine Microbiology, Düsternbrooker Weg 20, 24105, Kiel, Germany; Netherlands Institute of Ecology (NIOO/KNAW), NETHERLANDS

## Abstract

Molecular diversity surveys have demonstrated that aquatic fungi are highly diverse, and that they play fundamental ecological roles in aquatic systems. Unfortunately, comparative studies of aquatic fungal communities are few and far between, due to the scarcity of adequate datasets. We combined all publicly available fungal 18S ribosomal RNA (rRNA) gene sequences with new sequence data from a marine fungi culture collection. We further enriched this dataset by adding validated contextual data. Specifically, we included data on the habitat type of the samples assigning fungal taxa to ten different habitat categories. This dataset has been created with the intention to serve as a valuable reference dataset for aquatic fungi including a phylogenetic reference tree. The combined data enabled us to infer fungal community patterns in aquatic systems. Pairwise habitat comparisons showed significant phylogenetic differences, indicating that habitat strongly affects fungal community structure. Fungal taxonomic composition differed considerably even on phylum and class level. Freshwater fungal assemblage was most different from all other habitat types and was dominated by basal fungal lineages. For most communities, phylogenetic signals indicated clustering of sequences suggesting that environmental factors were the main drivers of fungal community structure, rather than species competition. Thus, the diversification process of aquatic fungi must be highly clade specific in some cases.The combined data enabled us to infer fungal community patterns in aquatic systems. Pairwise habitat comparisons showed significant phylogenetic differences, indicating that habitat strongly affects fungal community structure. Fungal taxonomic composition differed considerably even on phylum and class level. Freshwater fungal assemblage was most different from all other habitat types and was dominated by basal fungal lineages. For most communities, phylogenetic signals indicated clustering of sequences suggesting that environmental factors were the main drivers of fungal community structure, rather than species competition. Thus, the diversification process of aquatic fungi must be highly clade specific in some cases.

## Introduction

For a long time, fungi were thought to have negligible ecological functions in aquatic systems [1]. Only recently, it has been shown that aquatic fungal communities contribute to elemental cycles and mineralization processes [2]. These roles have been demonstrated for planktonic fungal communities [3–5], and for fungal communities of the marine subseafloor [6]. Differences in community structure result in different functional activities of the community that in return affect the functioning of the ecosystem. It is crucial to understand the community structure as a multi-player assemblage and to gain insights into the community composition, structure, and diversity [7,8].

Studies of aquatic fungal communities have benefited immensely from the development of molecular techniques. Thus, nowadays, information on fungal communities from a wide range of habitats exists, for example from deep-sea environments [9], hydrothermal vent ecosystems [10], coastal regions [3], anoxic regions [11], lakes [12,13], rivers [14] or associated to aquatic animals [15,16], plants [17,18] or algae [19]. In contrast to studies of terrestrial fungal communities, most of these studies targeted the 18S rRNA gene as marker region instead of the commonly used Internal Transcribed Spacers (ITS)–the “announced fungal barcode” [20].

The existing molecular information has rarely been used to get a more profound view on aquatic fungi. Currently, the sparseness of sequence data with contextual (meta) information poses a major obstacle for comparative studies and the construction of reference data sets. Although contextual data guidelines have been developed addressing this issue, most published data do not yet follow them [21]. Thus, essential contextual data has to be recovered by tedious manual screening of databases and publications. Here, fungal culture collections become important. They have the potential to provide high-quality sequences, which are combined with relevant and detailed contextual data [22].

In a first step, we generated a contextualized 18S rRNA gene sequence reference dataset for aquatic fungi. This dataset included all publicly available nearly full-length 18S rRNA gene sequences as well as sequences from the marine fungal culture collection of the GEOMAR Helmholtz Centre for Ocean Research Kiel (Germany). We tested our hypothesis that habitat-specific biomes exist for aquatic fungi using the created dataset. We included in our study phylogenetic-based pairwise community comparisons, analyses of the taxonomic composition and of phylogenetic signals.

## Materials and Methods

### Collection of aquatic fungal community studies

We used the “WEB OF SCIENCE” (http://apps.webofknowledge.com), “Google scholar” (http://scholar.google.com) and textbooks references to retrieve all published community studies containing fungal sequence data. The resources were screened using key words such as “community”, “marine”, “freshwater”, “fungi”, “eukaryotic” or a combination of these. Papers were screened for fungal sequence data. A study was deemed suitable for inclusion in our dataset if the following criteria were met: (i) published study and (ii) study detected at least 15 different fungal species or OTUs. All publications were additionally screened for the marker gene used, and this information was recorded. If several marker genes were used in the study, each marker gene was counted separately.

### Sequence collection and alignment

In June 2013, high-quality nearly full-length fungal 18S rRNA gene sequences were obtained from release 111 of the SILVA SSU Ref dataset [23] (released July 2012). Briefly, sequences in the SILVA Ref dataset were retrieved from the European Nucleotide Archive (ENA) release 111 (http://www.ebi.ac.uk/ena/) and aligned with the SILVA Incremental Aligner (SINA) [24]. The sequences were then subjected to the following quality criteria: (i) sequence length ≥ 1200 bp, (ii) alignment identity value above or equal to 70, and (iii) alignment quality above 50. Based on the manually curated SILVA taxonomy, this dataset contained 13,501 fungal sequences. All further steps sequence alignment refinement and phylogenetic analyses were carried out with the ARB software [25].

We identified a subset of 1,537 unique sequences with aquatic origins [26]. We verified that all sequences from publications older than mid 2013 were present in the SILVA dataset, if they fulfilled the quality criteria. We added 247 fungal 18S rRNA gene sequences from the KSMP Kiel (Kultur Sammlung Mariner Pilze Kiel) culture collection (GEOMAR Helmholtz Centre for Ocean Research, Kiel, Germany) to the aquatic subset, and also aligned them using the SINA aligner following manual refinement. All of these sequences originated from marine-derived fungal cultures, were ≥ 1,200 bp in length and contained less than 5 ambiguous bases. Cultivation of fungi and DNA extraction were carried out as described in Wiese *et al*. [27]. Specifically, PCR for amplification of fungal 18S rRNA gene fragments was performed using puReTaq Ready-To-Go PCR Beads (GE Healthcare, Solingen, Germany) with the primers NS1 (5'-GTA GTC ATA TGC TTG TCT C-3´) and FR1 (5'-AIC CAT TCA ATC GGT AIT-3') according to Gomes *et al*. [28]. PCR parameters were set to the following values: initial denaturation (8 min at 94°C), 35 cycles of primer denaturation (30 sec at 94°C), annealing (45 sec at 48°C), and elongation (3 min at 72°C) followed by a final elongation step (10 min at 72°C). Closest relatives of the sequences were identified by sequence comparisons against the National Center for Biotechnology Information (NCBI) GenBank database (http://blast.ncbi.nlm.nih.gov/Blast.cgi) using BLASTN [29]. Sequences from fungal strains obtained during this study were submitted to the GenBank database and were assigned accession numbers KJ939309 –KJ939313 and KM096133 –KM096374, respectively (S1 Table).

### Contextual data and trait analyses

We manually added structured contextual data to all sequences in this study [26]. This contextual data defines the environment where each fungal sequence originated. The new “environment” field allows EnvO-Lite controlled vocabulary with 20 different terms (http://obo.cvs.sourceforge.net/viewvc/obo/obo/ontology/environmental/EnvO-Lite-GSC.obo?revision=1.4&view=markup) [30]. These annotations are based on the contextual data fields like isolation source, title, and host that were already available in the SILVA SSU Ref dataset. Finally, sequences were assigned to seven EnvO-Lite defined habitat categories, namely animal-associated, aquatic, freshwater, hot spring, hydrothermal vent, marine, and sediment. The EnvO-Lite category “plant-associated” was exchanged for “plant/algae-associated”, as several sequences were derived from seaweed samples. Furthermore, two additional categories were created, namely seawater and detritus, as 17% of all sequences in our dataset originated from samples of these two habitat types [26]. Detailed information on habitat definition can be found in Table 1.

**Table 1:** Definition of the ten habitat categories used in this study. All categories are based on EnvO-Lite controlled vocabulary with the exception of “detritus” and “seawater”. The EnvO-Lite defined habitat “plant-associated” was extended to include algal samples as well.

Habitat category	Description
Animal-associated	A habitat that is in or on a living animal. Here "animal" denotes an individual of a species that is a sub-taxon of NCBITaxon:33208. The sequences of our dataset were derived exclusively from marine animals, with the exception of one.
Aquatic	A habitat that is in or on water. This category contains all sequences of our dataset that do not fall into freshwater or marine categories, such as peatlands with high amount of dissolved solids and low salt concentration.
Detritus	A habitat that is in or on death organic material. The sequences of our dataset were exclusively of marine origin, with the exception of one.
Freshwater	A habitat that is in or on a body of water containing low concentrations of dissolved salts and other total dissolved solids.
Hot spring	A spring that is produced by the emergence of geothermally-heated groundwater from the Earth's crust.
Hydrothermal vent	A fissure in the Earth's surface from which geothermally-heated water issues.
Marine	A habitat that is in a sea or ocean containing high concentrations of dissolved salts and other total dissolved solids (typically >35 grams dissolved salts per liter). This category contains all marine-origin sequences of our dataset, but could not be further assigned to a more specific category.
Plant/algae-associated	A habitat that is in or on a living plant or algae. This category contains all sequences of our dataset that were exclusively derived from marine plants and algae.
Seawater	Any water in a sea or ocean that is neither close to the bottom nor near the shore, and originating from the pelagic zone.
Sediment	Sediment is an environmental substance comprised of any particulate matter that can be transported by fluid flow and which eventually is deposited as a layer of solid particles\on the bed bottom of a body of water or other liquid. Sequences of our dataset originated from both marine and freshwater samples.

EnvO-lite definitions (http://obo.cvs.sourceforge.net/viewvc/obo/obo/ontology/environmental/EnvO-Lite-GSC.obo?revision=1.4&view=markup).

It was unfortunately not possible to further subcategorize e.g. seawater into horizontal zonation of seawater column, or sediment into marine vs. freshwater sediment due to poor contextual data. Sequences assigned to the categories detritus, animal- and plant/algae-associated were all derived from marine samples (except two sequences from detritus and animal-associated categories).

### Phylogenetic analysis

The original alignment was filtered using a 50% base conservation filter in the ARB software environment [25]. This filter left 1,562 informative alignment columns out of the complete alignment for phylogenetic analyses. Maximum likelihood tree reconstruction was performed with RAxML version 7.0.4 [31], applying the previously created base conservation filter, along with a termini filter to filter out any non-rRNA sequence overhangs. The GTRGAMMA model of DNA substitution has been chosen since the pinvar and the alpha parameter cannot be estimated independently [32]. RAxML was run using 100 rapid bootstrap inferences, using the bootstrap trees as starting trees. Eight choanoflagellate sequences were used as outgroup taxa, as choanoflagellates are one of the most closely related non-Fungi. The tree was edited with Inkscape, version 0.48 (http://inkscape.org).

### Phylogenetic affiliation of basal fungal lineages

As the majority of sequences from basal fungal lineages were environmental sequences and had erroneous taxonomic assignments, further analyses were carried out to resolve their taxonomy. Sequences that grouped with basal fungal lineages in the phylogenetic tree reconstruction step were subjected to three additionally analyses. Firstly, BLASTN was performed at the NCBI BLAST website with default parameters [29]. Secondly, the phylogenetic placement of these sequences was checked in the eukaryotic guide-tree of the SILVA SSURef 111 dataset. Finally, current literature was consulted for more information on taxonomic position. The reported taxonomic affiliations for basal fungal lineages reflect the combined results of these three approaches (S2 Table).

### Detection of differences in aquatic fungal communities

Fungal assemblages of the ten defined habitats (see 2.3) were analyzed in different ways. Firstly, the habitat-specific taxonomic compositions were inspected by subsampling sequences to the sequence number of the smallest community using the QIIME pipeline [33] (hot spring samples were excluded due to low sequence number). Taxonomic charts were constructed by clustering the subsampled sequences into operational taxonomic units based on 99% sequence identity (OTUs_0.01_), a commonly used threshold for clustering fungal 18S rDNA sequences [34]. Secondly, fungal assemblages were tested for significant differences between habitats. Thus, sequences were pooled according to their assignment to different habitat categories (see 2.3). These habitat–pooled sequences were subjected to a Significance test based on weighted UniFrac values [35]. The weighted UniFrac metric is the phylogenetic Kantorovich-Rubinstein distance between two sequence distributions in a phylogenetic tree [36]. It is, therefore, a mathematically well-defined metric for significant differences between communities. Permutation tests were carried out with the FastUniFrac suite [35] implemented in the Galaxy platform [37,38]. Testing involved 1,000 Monte Carlo steps (the maximum number permitted by the Galaxy server). To check for convergence we repeated each test forty times. We assumed significant difference if the Bonferroni corrected p-value was <0.05 each time. Finally, we tested for significant phylogenetic differences between sub-communities in each habitat. To this end we calculated weighted Unifrac distances for all pairs of sub-communities in each habitat that contained at least four sub-communities with 25 sequences or more. Proof of general significance (P<0.05) of phylogenetic beta-diversity between habitats was deduced by running an one-way ANOVA implemented in R version 3.1.0 [39]. We subsequently identified significantly different pairs of habitats using the Scheffé *post hoc* test (P<0.05).

### Phylogenetic signals

Phylogenetic signals of different habitats were tested with the software PHYLOCOM v 4.2 [40], using the COMSTRUCT function. Phylogenetic relatedness was estimated via the net relatedness index (NRI; the average phylogenetic distance within a community) and the nearest taxa index (NTI; the average phylogenetic distance to the closest relative in a community). Phylogenetic signals can indicate either overdispersion or phylogenetic clustering of taxa in the communities. Thus, NRI shows how many taxa in a community are dispersed/clustered over the whole phylogenetic tree, while the NTI indicates dispersal/clustering on lower taxonomic levels. As a null model the “–m 2” function was chosen, to account for species in the phylogenetic tree lacking contextual data. Here, samples of the null communities were created with species randomly drawn from all taxa present in the phylogenetic tree. The number of randomizations was set to 9999. A p-value of P<0.05 against the random distributions was considered to indicate a significant phylogenetic signal. In a last step, NODESIG analyses were run to identify the clades in the phylogenetic tree that led to the detection of phylogenetic signals.

## Results and Discussion

### What 18S rRNA gene sequences can tell us about aquatic fungal communities

Fungal community surveys in aquatic and terrestrial realms have proceeded at different rates and depths, with surveys of aquatic fungi often lagging behind. This trend has recently changed and there is now more interest in understanding aquatic fungal communities. Several studies have linked aquatic fungi with ecosystem functioning [41]. The kindled interest is further fueled by the biotechnology industry. Enzymes from aquatic fungi show distinct physiological characteristics, such as high salt tolerance or barophilicity, which are highly prized in industrial applications [42].

In the past, studies of aquatic fungi relied on microscopic observations [43]. With the advent of the molecular era, these studies also started employing Sanger-sequencing like culture-trapped fungal isolates [19,44,45] or environmental clone libraries [46]. Aquatic fungal-specific surveys applying next generation high-throughput sequencing approaches just started in 2012 with only a handful of data currently available [13–15,47–50].

**Fig 1:**
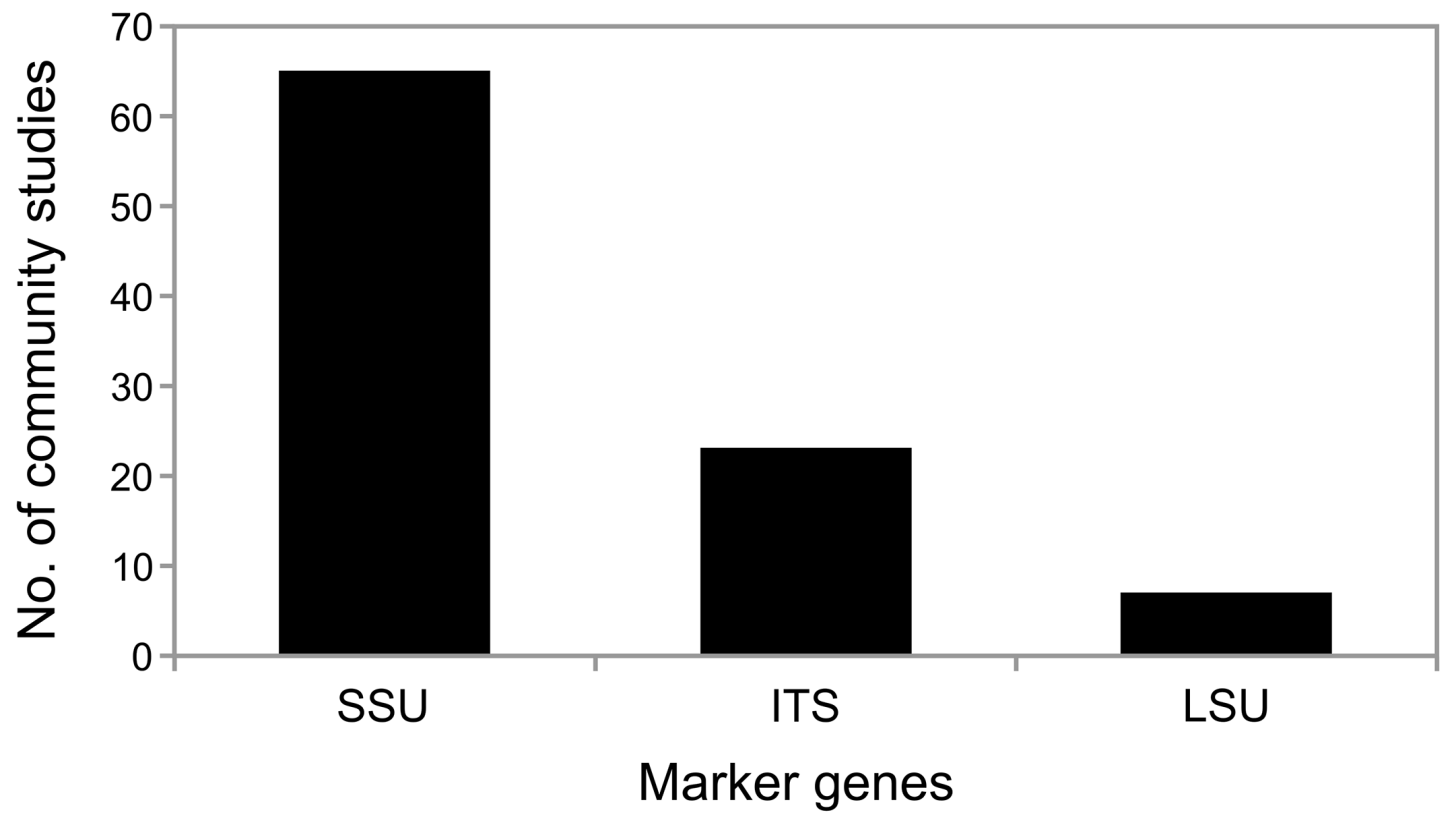
Number of aquatic fungal community studies and the different fungal marker genes targeted. For the summary all community studies containing fungal sequence information were counted, which targeted one of the following marker genes: ITS, LSU or SSU.

Most often, aquatic fungi are studied as part of eukaryotic or protistan communities [51–54], while molecular studies using fungal-specific primers are less common [48,55]. Therefore, the majority of sequence data on aquatic fungi is 18S rRNA gene sequences (Fig 1). The ITS regions perform poorly in inferring phylogenies [56] and thus, only 352 of the high-quality fungal ITS sequences derived from aquatic systems in the UNITE database [57] can be assigned to any taxonomic level (https://unite.ut.ee/, accessed January 2015). However, as the aquatic realm is a rich source of novel fungal species and groups [58,59], proper phylogenetic inference is needed to resolve their placement within the fungal tree of life. Thus, current aquatic fungal-specific studies use a double marker gene approach. They target the 18S rRNA gene and the ITS regions [48,60], which results in a deeper taxonomic resolution of the community [47].

In addition to improve taxonomic classification, phylogeny-based community analyses give community surveys another flavor. They enable the inspection of the community from an evolutionary perspective [35]. It is possible to test the relevance of phylogenetic clades for the functioning of a community [61], or the lineage specific evolution as a response to specific factors [40]. Hierarchical clustering methods forming OTUs on an arbitrary threshold fail in this regard.

In summary, the accumulated “full-length”18S rRNA sequence data on aquatic fungi should not be disregarded. Instead, it should be viewed as a valuable resource leading to a better understanding of aquatic fungal communities.

### A full-length 18S rRNA gene dataset for aquatic fungi

**Fig 2:**
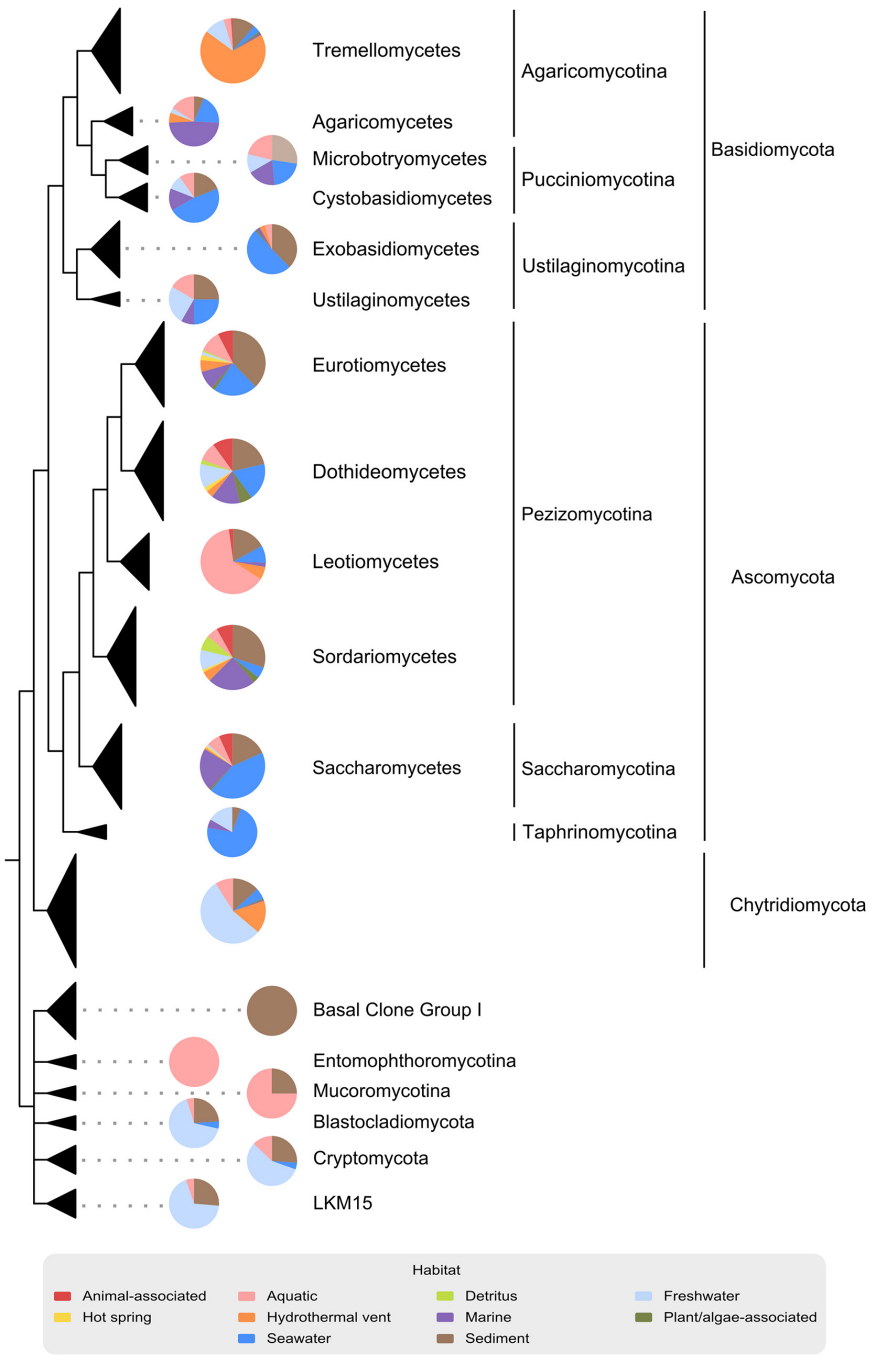
Schematic diagram of the phylogenetic tree using 18S rRNA gene sequences from aquatic fungi. Sequence data were collected from the SILVA dataset and the KSMP-Kiel culture collection. Thickness of taxonomic triangles is proportional to the sequence amount of each taxonomic groups. Piecharts beside triangles show group-specific sequence assignments to the habitat types.

Our dataset is the first comprehensive in-depth survey of published nearly full-length 18S rRNA gene sequence data on aquatic-derived fungi (S1 Fig) and contains 1,784 fungal sequences [26]. Consistent with literature [58,62], *Ascomycota* was the largest group (Fig 2). The three main classes of *Ascomycota*—*Eurotiomycetes*, *Dothideomycetes* and *Sordariomycetes* (Fig 2)—are well known and dominant marine fungal lineages [63]. Interestingly, a high proportion of *Basidiomycota* sequences were also found in our dataset, with the majority being yeast fungi (Fig 2, S1 Fig). Recent studies on isolated *Basidiomycota* reported less than 100 species isolated from aquatic sources [62,64], but molecular data suggest that *Basidiomycota* diversity must be much higher in aquatic environments [9,17]. *Chytridiomycota* formed the third largest sequence group in our dataset. Furthermore, seven additional phyla/subphyla of basal fungal lineages were discovered. Of those, the recently described *Cryptomycota* [65] formed the largest sequence group (Fig 2; S2 Table). However, a reliable statement about the proportion of these seven groups cannot be made. Partly due to PCR primer bias, sequences associated with basal fungal lineages are often not detected in molecular surveys [11]. For instance, *Cryptomycota* normally accounts for only 0.02–4.5% of total sequence reads [66], and additionally, it is generally difficult to map these sequences to reference clades.

In order to investigate how the habitat affects fungal community assemblages, we had to link the sequence data with contextual data on sampling location (S1 Fig) and habitat type (Fig 2, Table 1). Most sequences were only sparsely annotated and were not following the “Minimum Information about a Marker Gene Sequence” requirements [21]. We added all relevant contextual data by manually screening databases and publications. Thus, our dataset is a valuable resource for further ecological studies, as well as a high quality reference dataset [26].

Using the modified EnvO-Lite controlled vocabulary, sequences could be categorized into ten different habitat categories (Table 1). The sequences were assigned to both marine and freshwater categories, as well as to organism-associated categories (Fig 2). Further, sampling locations showed global distribution over six continents and eight oceans (S1 Fig) [26].

### Fungal community structure across aquatic habitats

#### 1.1.1 Fungal communities in freshwater environments

**Fig 3:**
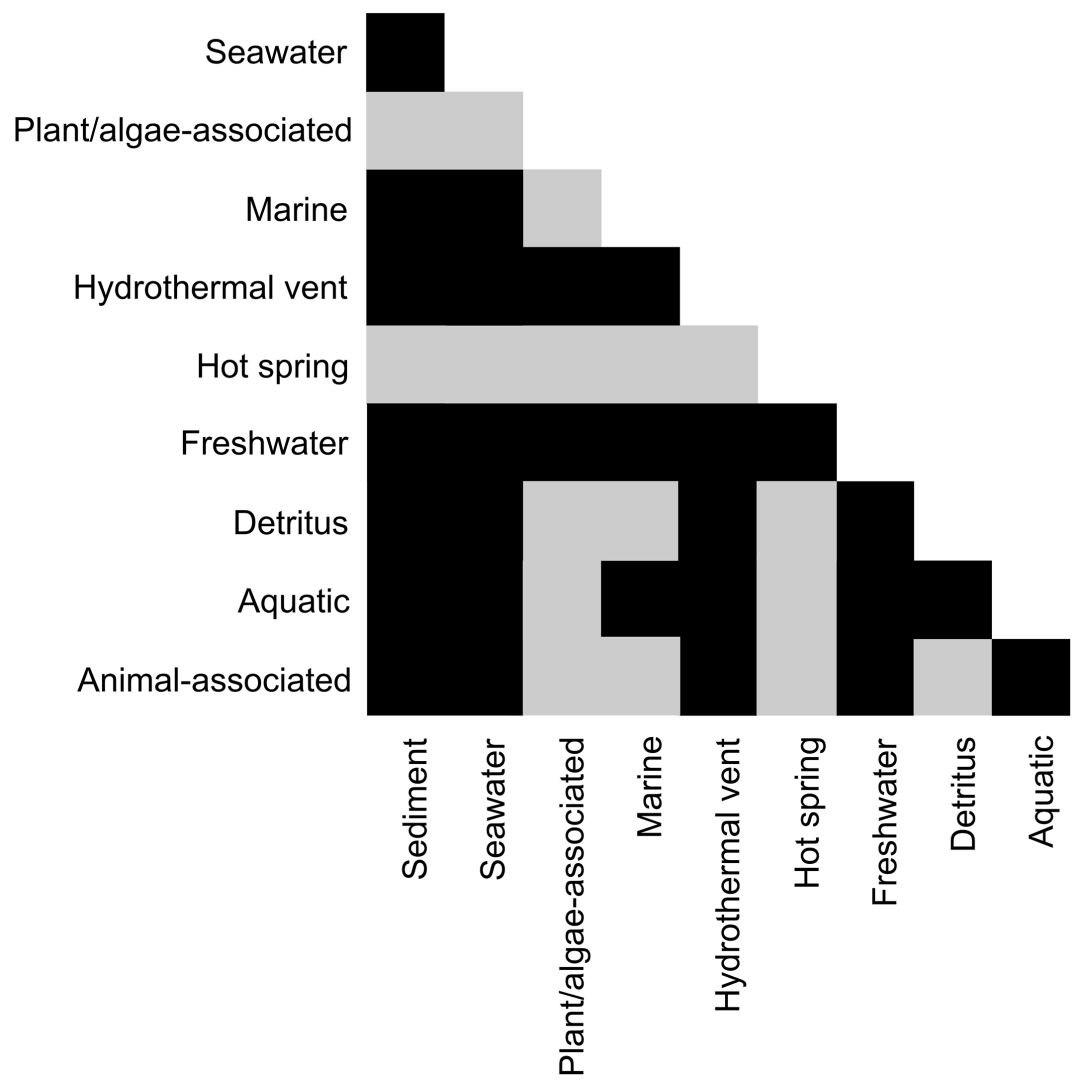
Impact of habitat on fungal assemblages. Pairwise overlap of fungal assemblages according to habitat types; significant differences (P < 0.05) shown in black.

**Fig 4:**
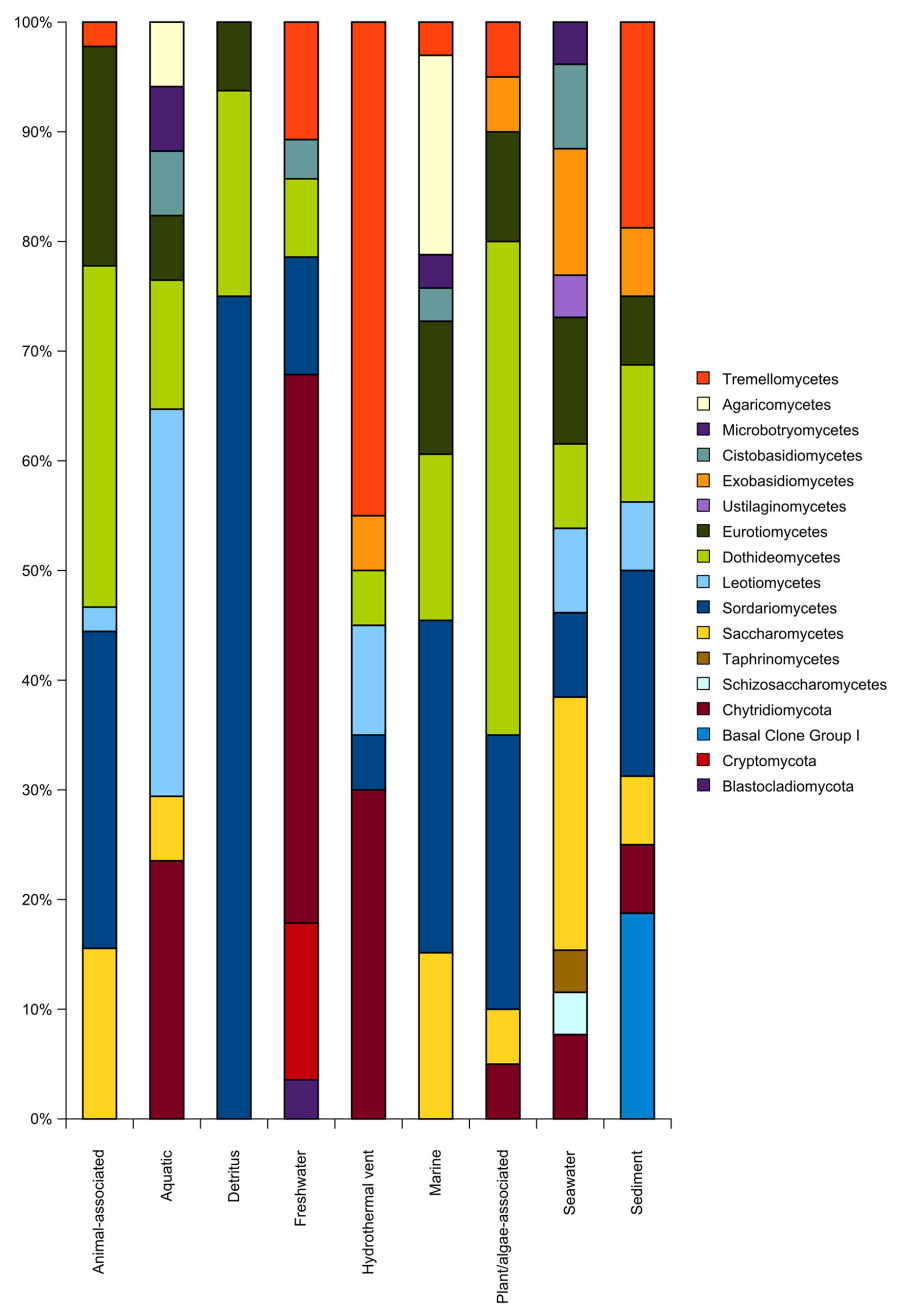
Taxonomic assemblages of different habitat types. Subsampled assemblages inferred from clustering sequences at a 99% similarity level. OTUs_0.01_ counts were calculated for all habitat categories at order level and for basal fungal lineages at phylum/subphylum level. (Hot spring excluded due to low sequence number).

**Fig 5:**
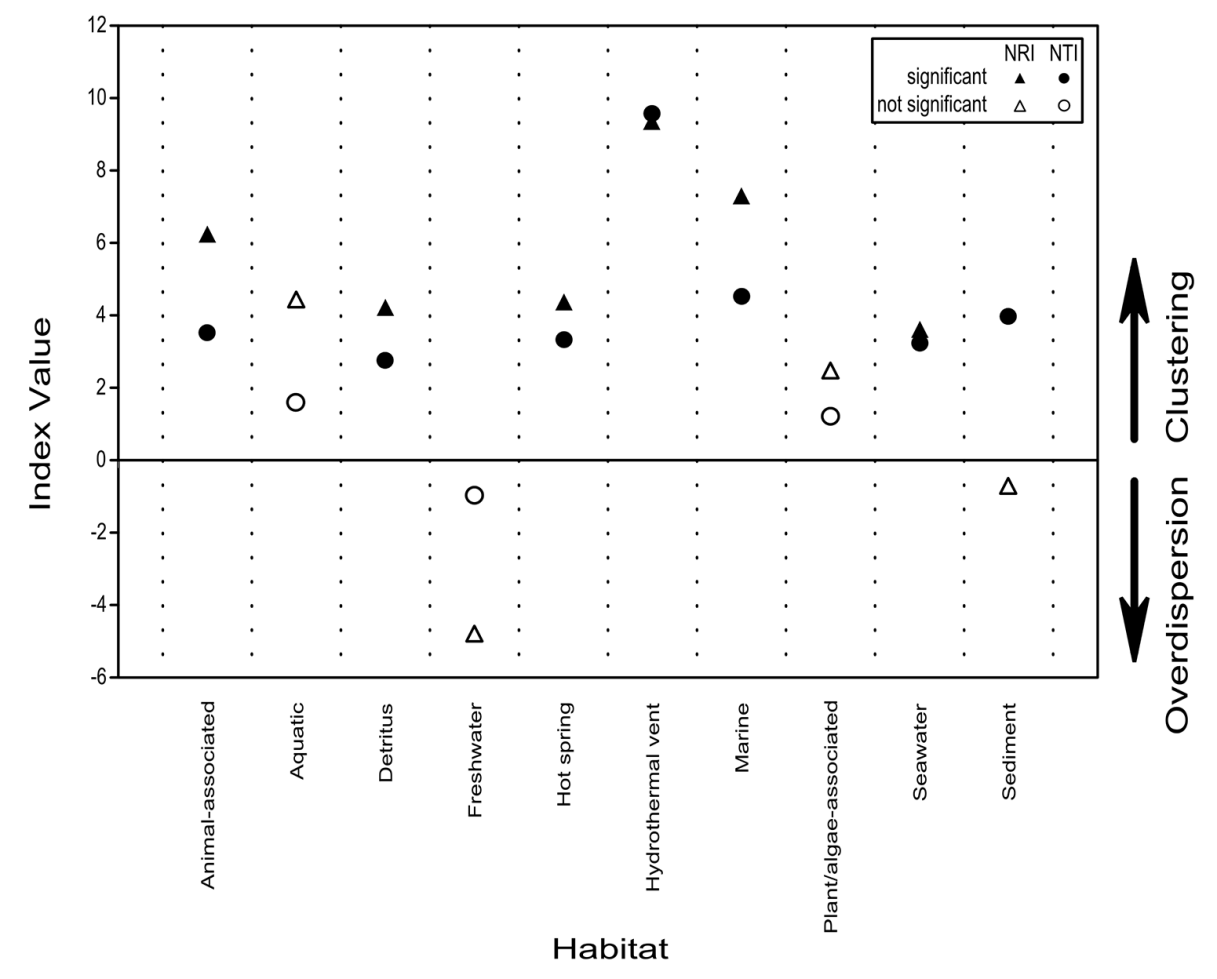
Phylogenetic signal analyses of aquatic fungal taxa present in different habitats. Net Relatedness Index and Nearest Taxa Index demonstrate clustering or overdispersion of fungal taxa in a habitat over the whole pool of phylogeny or within particular terminal clades, respectively. Comparison of observed data against randomly generated samples, number of generations = 9999.

**Fig 6:**
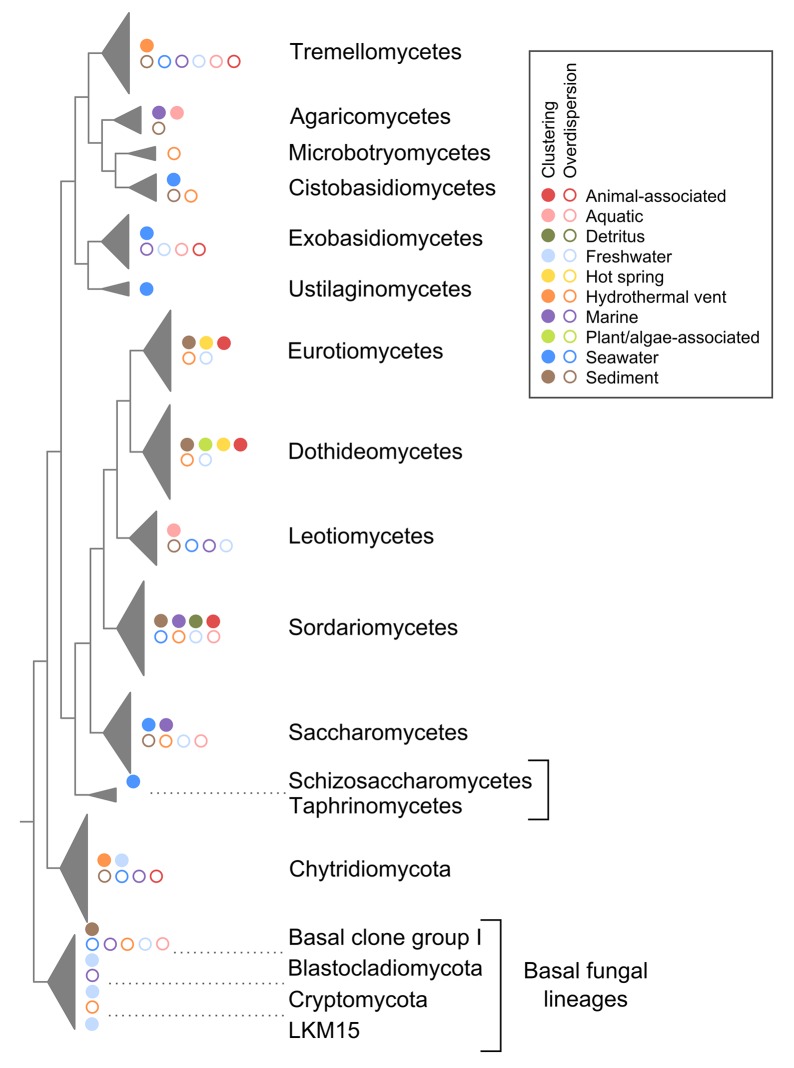
Positions of phylogenetic clustering or overdispersion within the phylogenetic tree tested by NODESIG analyses. Only significant results are shown. Full circle, clustering signal; open circle, overdispersion.

Freshwater fungal community structure differed significantly from all other habitat communities (Fig 3). This is similar to what has been observed for marine and freshwater microorganisms, which usually group in distinct marine and freshwater phylogenetic clusters [67]. Nearly 70% of all OTUs_0.01_ clustered in basal fungal lineages (Fig 4) and most of them belonged to the *Chytridiomycota*, which is known to be dominant in most freshwater samples [68,69]. *Chytridiomycota* play various roles as parasites [41] or in decomposing processes [5]. The other 30% of OTUs_0.01_ clustered equally in *Asco-* and *Basidiomycota*. We observed a tendency of taxonomic overdispersion in the freshwater habitat, mainly caused by these two phyla (Figs 5 and 6). Compared to other habitats, fungal assemblages of freshwater showed the highest diversity on the phylum level (Fig 4, subsampled data). The total amount of fungal sequences assigned to freshwater habitats clustered in several different fungal classes and subclasses (Fig 2). In contrast to other systems, freshwater systems are rich in carbon sources and structures, due to high terrestrial carbon input. Furthermore, freshwater systems are dynamic systems due to their physical nature, which degrade, sequester or translocate carbon [70]. In the pelagic zone of freshwater ecosystems, decreased amounts of soil organic carbon (C) associated with nutrient release from soil-microorganisms greatly influences the C dynamics, as well as the nutrient balance [71,72]. Fungi play essential roles in these freshwater nutrient processes [73]. As *Chytridiomycota* lack the ability to degrade cellulose [74], pre-processing of substrates by *Ascomycota* and *Basidiomycota* is essential to allow the colonization and use as a feeding source by other organisms [73,75]. Although several freshwater *Ascomycota* species have been intensively studied, about 70% of them were only reported once [62], explaining the tendency of overdispersion of higher fungi in our dataset.

Taxonomic diversity (Fig 4) and the observed phylogenetic diversity of the subsamples in our dataset (S2 Fig) could also be explained by the diverse ecology of freshwater systems. They can be divided into different groups, which belong either to the lentic systems such as lakes or ponds, or to lotic systems with a continuous water flow. However, even among lakes, environmental conditions such as physicochemical parameters are diverse [76]. Diverse environmental conditions within individual systems lead to distinct demands on fungal adaption and dispersal strategies [77].

#### 1.1.2 Fungal communities in sediment environments

The NRI value of fungal assemblages from sediments was negative (Fig 5). This indicates a tendency (though not significant) of overdispersion at higher taxonomic levels (Fig 6). Thus, several groups of *Ascomycota*, *Basidiomycota*, *Chytridiomycota*, and other basal fungal lineages were reported. None of these formed a dominant group on higher taxonomic rank levels (Figs 2 and 4). Sediments show a large surface area, which is susceptible to abrasion. A key step for microorganisms to survive in this environment is their ability to attach to protected areas of grains or particles [78]. Additionally, fungi have to adapt to sediment innate conditions [6], such as a sharp oxygen gradient, particular nutrient and organic matter absorption capacity, or selective entrainment transport [79,80]. Hence, fungal assemblages of sediments were significantly different than assemblages of nearly all other habitat categories (Fig 3). The environmental pressure especially affected the phylogenetic diversity of *Eurotiomycetes*, *Dothideomycetes*, *Sordariomycetes* and the Basal Clone Group I, as these groups showed significant phylogenetic clustering (Fig 6). Phylotypes of the Basal Clone Group I have so far only exclusively been detected with molecular tools and mainly been found in sediment samples [81–83], with few exceptions [84,85]. Thus, it is unclear if the phylogenetic signal of the Basal Clone Group I is due to environmental pressure, or due to incomplete species data on members of this group. In contrast, *Sordariomycetes* and *Dothideomycetes* are among the largest groups of marine *Ascomycota* (Fig 2) and have a broad spectrum of morphology and functionality [63], where only specific subclades seem to be adapted to life in sediments (S1 Fig).

#### 1.1.3 Fungal communities in seawater environments

The fungal assemblages of seawater (pelagic zone) showed significant phylogenetic clustering (Fig 5). As the larger groups within OTUs_0.01_ counts (Fig 4), yeast fungi of *Basidiomycota* and *Ascomycota* were mainly responsible for this signal (Fig 6). These results suggest that unicellular fungal forms dominate not only deep oceans [9], but also seawater. On the other hand, filamentous fungal forms have been reported to dominate locally [3] or seasonally [4]. They are also positively correlated with phytoplankton abundance, or associated with swimming food sources from detritus particles to nematodes [86]. In comparison to other habitat types, the fungal assemblage of seawater was the most diverse one with respect to the represented fungal richness on class level. One possibility could be intra-habitat specific differences as found for some sub-communities in our dataset (S2 Fig). Geographical and/or environmental barriers might exist. For example, carbon source fluxes are known to have a priming effect in freshwater ecosystems [87], and they could act in a similar manner in seawater as well. Local scale differences in fungal communities, e.g. in pelagic depth zones [88], have also been reported. However, more intensive sampling is needed to support such hypotheses and to determine the factors that impact the geographical dispersal of fungi in seawater at different scales.

#### 1.1.4 Fungal communities in marine hydrothermal vent systems

The hydrothermal vent category was the only habitat with a dominant *Basidiomycota* fraction. About 50% of OTUs_0.01_ were assigned to this phylum (Fig 4). *Basidiomycota* taxa clustered mainly in clades known to contain yeast fungi (S1 Fig), which is the dominant fungal form in the deep sea [9]. They were also responsible, besides *Chytridiomycota* taxa, for the high NRI and NTI values. High NRI and NTI values indicate whole clade, as well as species exclusion (Figs 5 and 6) [89]. In hydrothermal vent regions, adaptation to high temperature is necessary. However, only a handful of described fungal species are recognized to be thermophilic or are able to adapt to temperatures above 45°C [90]. This could be the reason why we did not find a significant difference between fungal assemblages of hydrothermal vents and hot spring habitats (Fig 3). However, habitats with high temperatures are heterogeneous in terms of their physical and chemical characteristics [90]. The extremely high phylogenetic signal observed for hydrothermal vents, in contrast to hot spring habitats (Fig 5), suggests that environmental conditions other than temperature generate an extreme selection pressure in hydrothermal vents, where only highly adapted fungi can diversify. Our analyses suggest that such highly adapted fungi are found particularly within *Tremellomycetes* and *Chytridiomycota* (Fig 6).

#### 1.1.5 Fungal communities associated with marine plant/algae

The fungal community composition found in marine plants/algae did not differ significantly from other habitats, except for freshwater and hydrothermal vent (Fig 3) and was dominated by *Eurotiomycetes*, *Sordariomycetes* and *Dothideomycetes* (Fig 4). These three *Ascomycota* groups could be detected in nearly all other habitat types (Fig 4). *Dothideomycetes* form the largest group within the phylum *Ascomycota* and exhibits a high ecological diversity [91]. Species belonging to this class have dramatically different genome sizes, and functional capabilities of individual species are highly variable [92], probably reflecting the wide spectrum of ecological adaptation in this fungal group.

The life cycles of plant-colonizing fungi often include stages independent of the host [43]. Furthermore, transmission of plant/algae-associated fungi may include spore dispersal, release of hyphal fragments, and passive distribution of infected or dead plant tissue [93]. These may be the reasons why marine plant/algae-associated fungal taxa were also detected in other marine habitats (S1 Fig) resulting into low number of significant differences in fungal assemblages (Fig 3). Our results suggest similarities between aquatic plant/algae-associated fungi and terrestrial root-colonizing fungi, which are detectable for a long time in soil prior to root infection [94]. In contrast to our findings, bacterial communities associated with the green algae *Ulva australis* are significantly different from communities sampled in the surrounding seawater [95]. Whether only one or both of these scenarios are plausible for aquatic fungal communities associated with plant/algae has to be verified in future studies.

#### 1.1.6 Fungal communities of marine detritus

All fungal sequences of our dataset assigned to detritus habitat clustered exclusively in *Eurotiomycetes*, *Sordariomycetes*, and *Dothideomycetes* (Fig 4). These three fungal classes are the sources of diverse industrially-important enzymes or bioactive compounds [96]. The process of decomposition is very complex and demands specific enzymatic activities. These activities were detected within various functional groups of fungi, but not for all species of those groups [97]. Additionally, fungal elemental composition with respect to the available nutrients also seems to be an important benefit for fungi in this habitat type. Fungi with no elemental homeostasis have a competitive advantage over bacteria on nutrient-depleted detrital resources [98], explaining the limited taxonomic diversity and the phylogenetic clustering in detritus samples (Figs 4 and 5, S1 Fig).

## Concluding Remarks

The most widely used marker gene of aquatic fungi in terms of available sequence amount is the 18S rRNA gene (Fig 1). Thus, the evaluation of existing 18S rRNA gene sequence data promises a more profound understanding on aquatic fungal communities. As the 18S rRNA gene sequence can be used for a phylogenetic evaluation apart from hierarchical clustering analysis of the fungal community, it can serve as a highly valuable complementary resource to ITS, providing deeper insights into fungal life in aquatic systems.

Here, we provide the first comprehensive dataset of nearly full-length 18S rRNA gene sequences of aquatic fungi with a rich set of manually curated contextual information, which can be used as a reference dataset including a phylogenetic tree [26]. *Ascomycota* are prevalent in the dataset (Fig 2). However, the observed *Basidiomycota* sequence richness and their distribution among diverse habitats (Fig 2) suggest a hitherto underestimation of this phylum in aquatic environments.

This dataset allowed us to show that habitat-specific biomes exist for aquatic fungal communities, as fungal assemblages of 28 out of 45 pairwise habitat comparisons were significantly different (P<0.05) from each other (Fig 3). Freshwater fungal assemblages differed to all other fungal assemblages and were dominated by the lineages of basal fungi. In contrast, the assemblage of the plant/algae habitat only differed significantly from two other assemblages showing that fungi associated to plants/algae are present in the surrounding habitats prior to host colonization.

Phylogenetic signals revealed several cluster effects suggesting that selection pressure within habitats was driven mainly by environmental factors and not by species competition (Fig 5). Thus, although no true environmental marine clade has yet been found [58], diversification processes of fungi in marine systems must have been highly clade specific in some cases.

As aquatic fungal community structures vary to a great extent with habitats, fungal species in aquatic environments seem to be very specific to their habitat, rather than being generalists. A large number of unknown fungal species are regularly detected in other studies [58], which further supports this idea.

Our combined dataset gives the first insights into general patterns of aquatic fungal communities. However, more in-depth aquatic fungal community surveys are urgently needed in order to achieve a more exhaustive perspective on fungal life in aquatic systems.

## Supporting Information

S1 Fig Subsections of the Maximum Likelihood tree of fungal 18S rRNA gene sequences. The tree was rooted with sequences of eight species of the *Choanoflagellates*. Branch support was estimated with 100 bootstrap calculations. Only bootstrap values ≥ 50% are indicated. Sequences derived from the KSMP-Kiel culture collection are printed in bold. Color code, contextual data: colored background of fungal taxa, habitat occurrence of fungal taxa; colored lines beside sequence full name, geographical origin of fungal taxa. Subcategories of the geographical origin displayed as letter to the right of the colored stripe. *, wrong full name annotation of sequence.
(PDF)

S2 Fig Phylogenetic distances between sub-communities of diverse habitat types. Length of boxes shows variation of phylogenetic distances inferred from pairwise sample comparisons. Black lines, median value. Different letters indicate significant differences (P<0.05) as determined by one-way ANOVA test followed by a Scheffé *post hoc* test. Number of sub-communities/habitat: four (except sediment: seven).
(PDF)

S1 Table Information on sequences of the KSMP-Kiel culture collection used for this study. After inspecting all BLASTN hits, sequences were assigned to a submission name.
(PDF)

S2 Table New taxonomic assignment of sequences of basal fungal lineages. Fungal sequences were subjected to BLASTN analysis and checked for their taxonomic placement in the eukaryotic guide-tree of the SILVA release 111. Sequences were classified depending on combined results from the methods mentioned above, as well as literature searches.
(PDF)
