# Supplementary material for: Identification of Habitat-Specific Biomes of Aquatic Fungal Communities Using a Comprehensive Nearly Full-Length 18S rRNA Dataset Enriched with Contextual Data
Source: PLoS One. 2015 Jul 30;10(7):e0134377. doi: 10.1371/journal.pone.0134377 (PMC4520555; doi:10.1371/journal.pone.0134377)

Supplementary Figure S1: Maximum Likelihood tree of fungal 18S rRNA gene sequences. The tree was rooted with sequences of eight species of the *Choanoflagellates*. Branch support was estimated with bootstrap method. Only support values $\geq 50\,\%$ are indicated. Sequences derived from the KSMP-Kiel culture collection are printed in bold. Color code, contextual data: colored background of fungal taxa, habitat occurrence of fungal taxa; colored lines beside sequence full name, geographical origin of fungal taxa. Subcategories of the geographical origin displayed as letter to the right of the colored stripe. $*$, wrong full name annotation of sequence.

Figure 1: **Subsection of *Tremellomycetes***

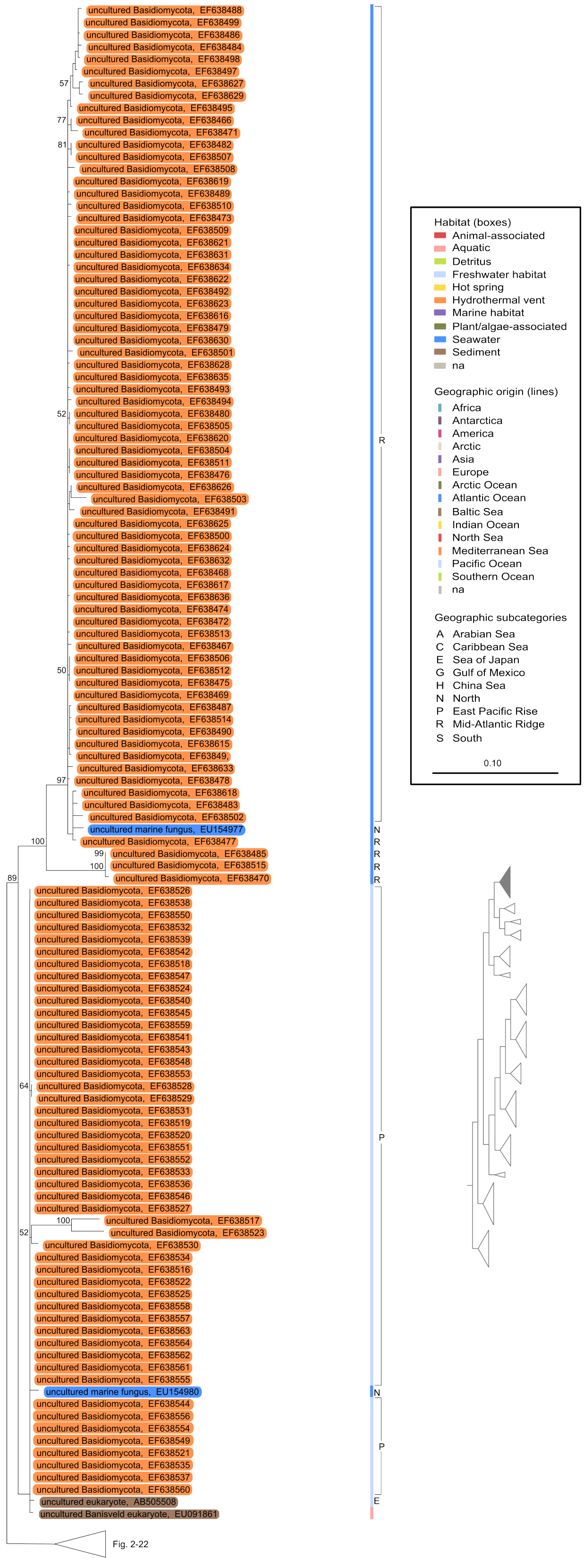

Figure 2: **Subsection of *Agaricomycotina***

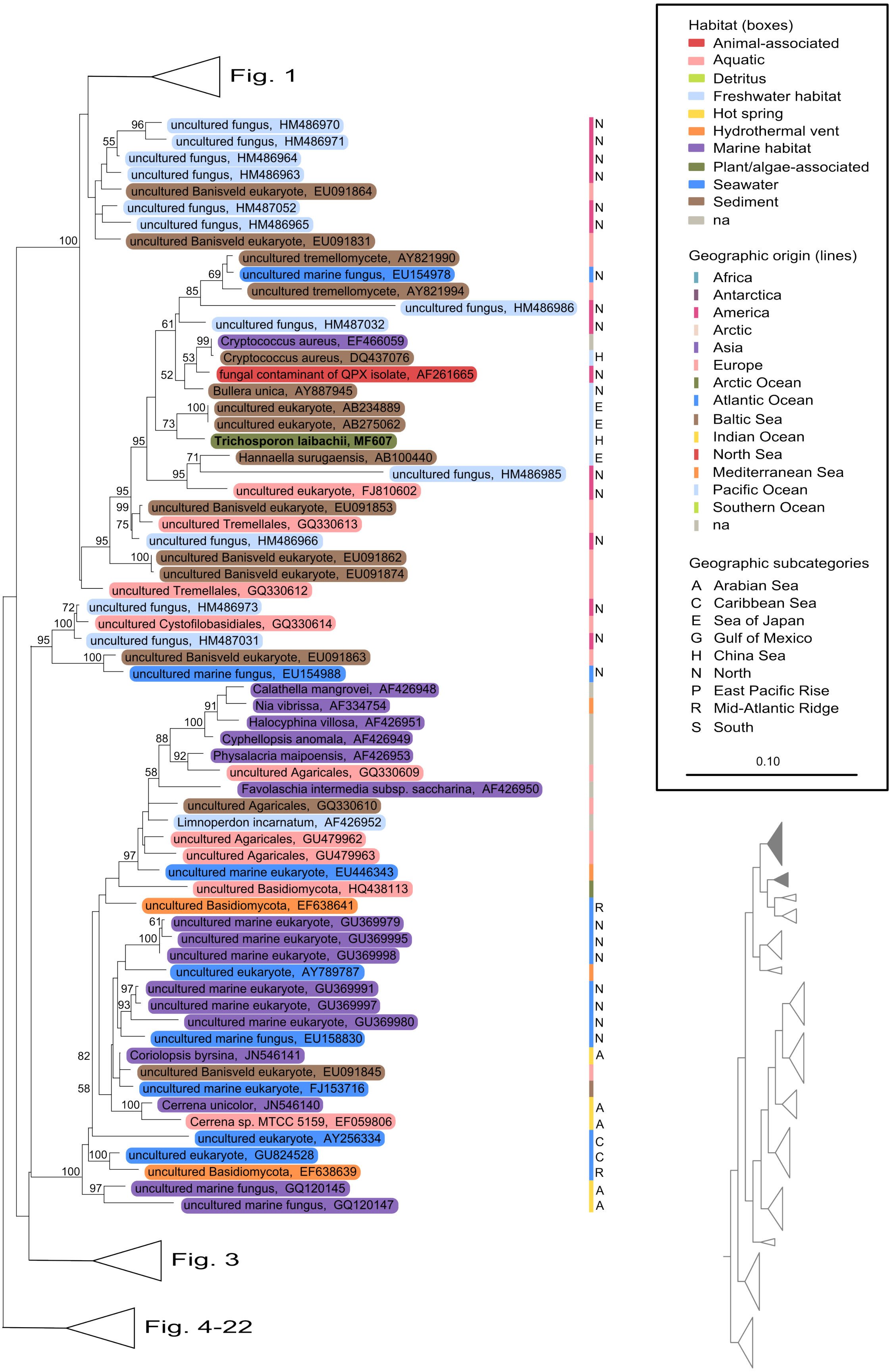

Figure 3: **Subsection of *Pucciniomycotina***

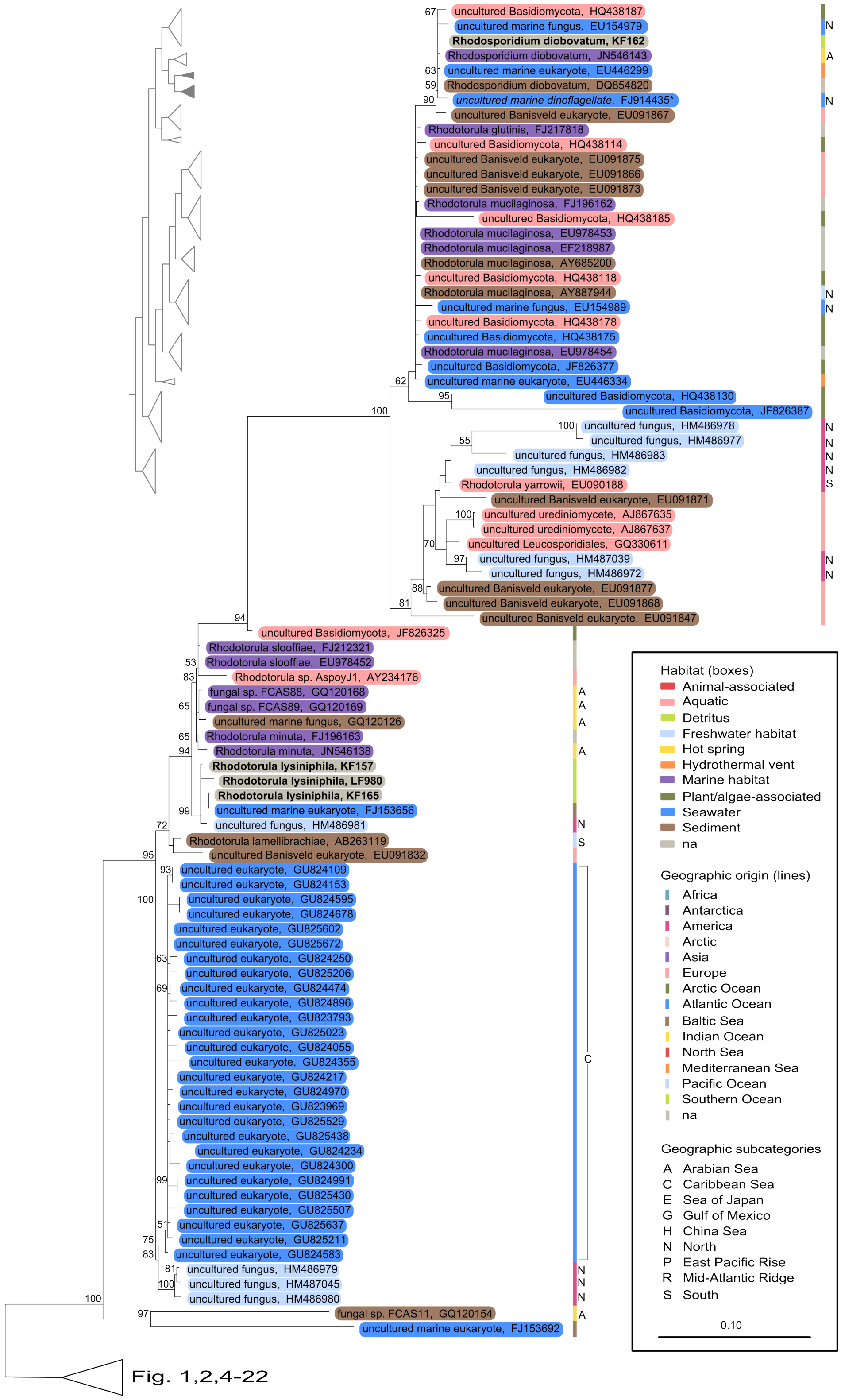

Figure 4: **Subsection of *Exobasidiomycetes***

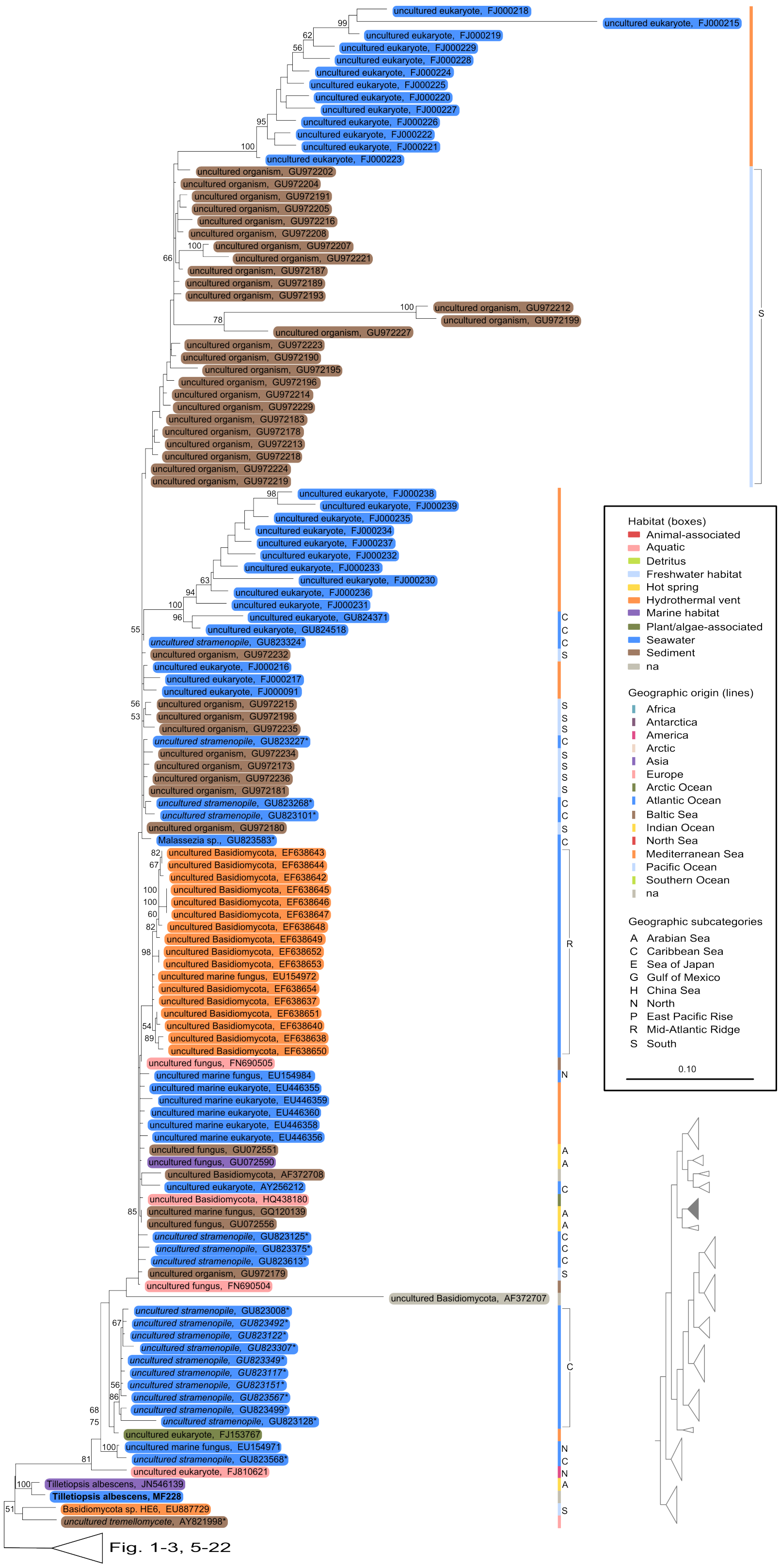

Figure 5: **Subsection of *Ustilaginomycetes***

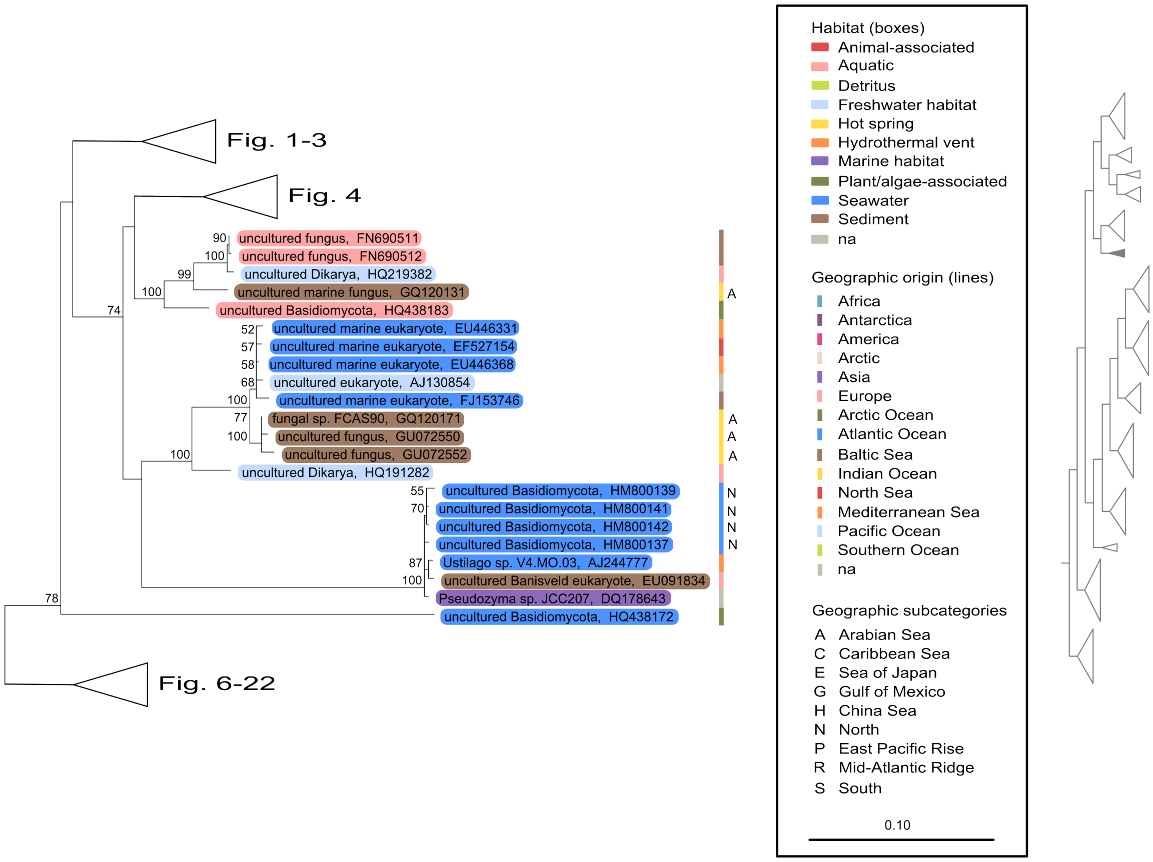

Figure 6: **Subsection of *Eurotiomycetes***

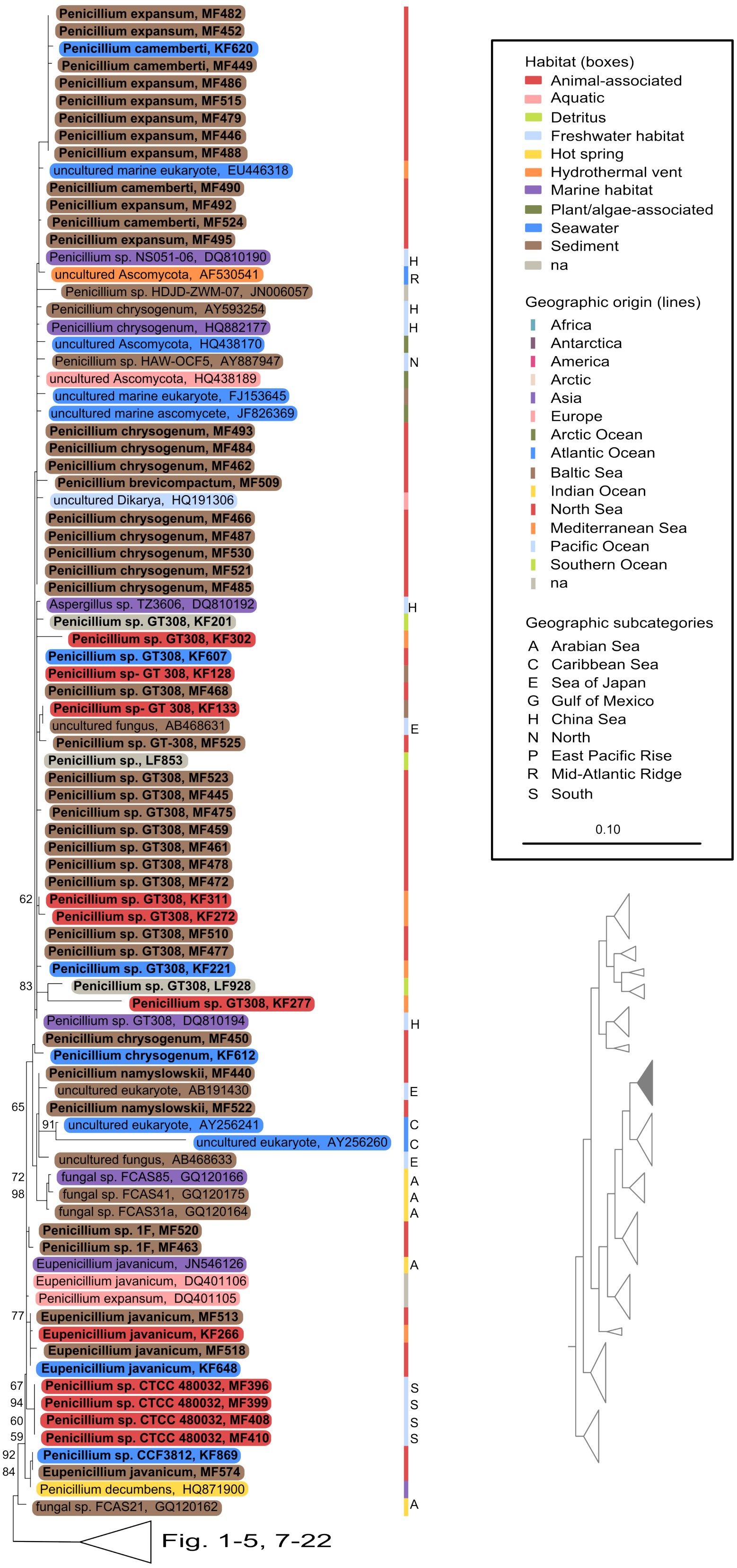

Figure 7: **Subsection of *Eurotiomycetes***

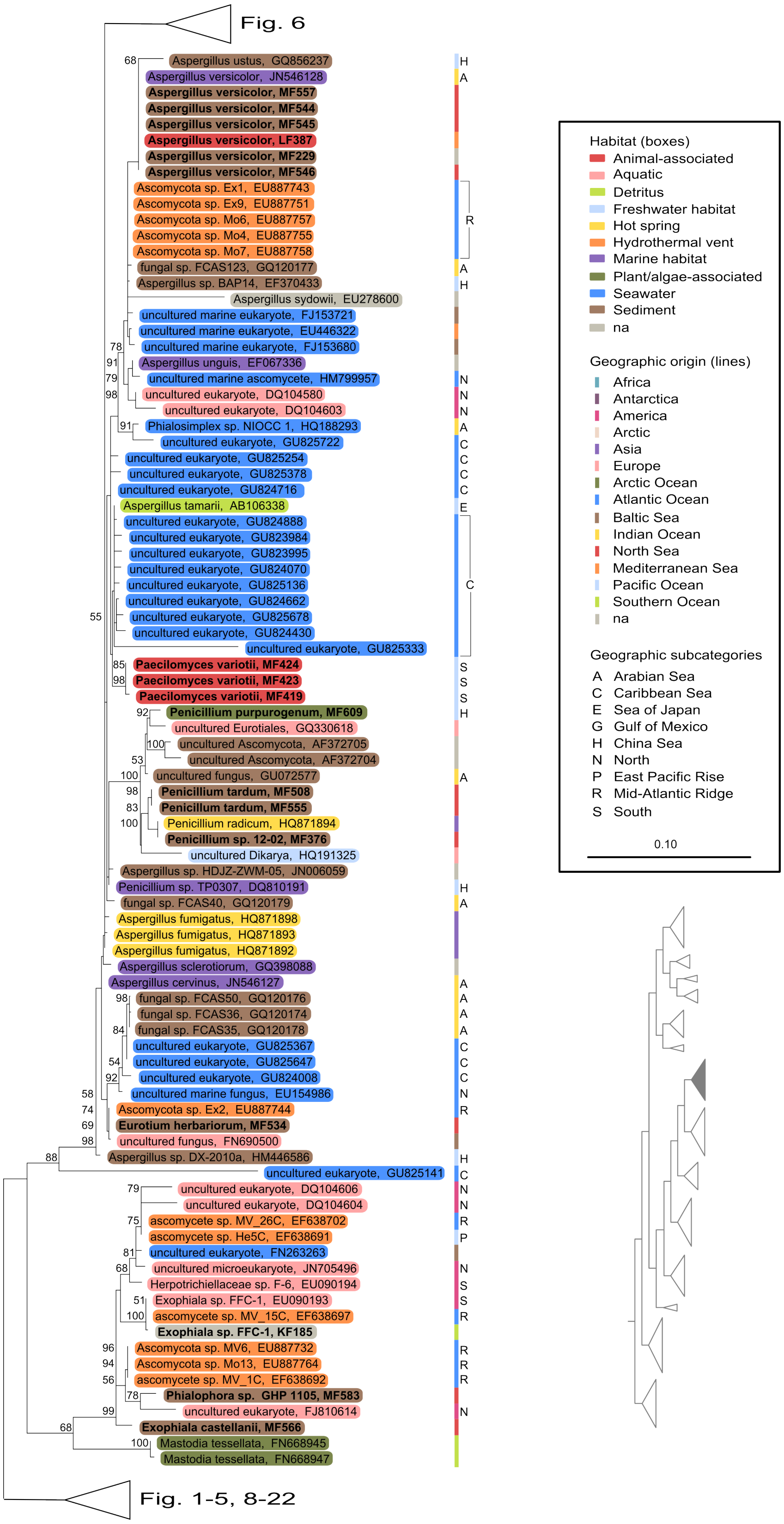

Figure 8: **Subsection of *Dothideomycetes***

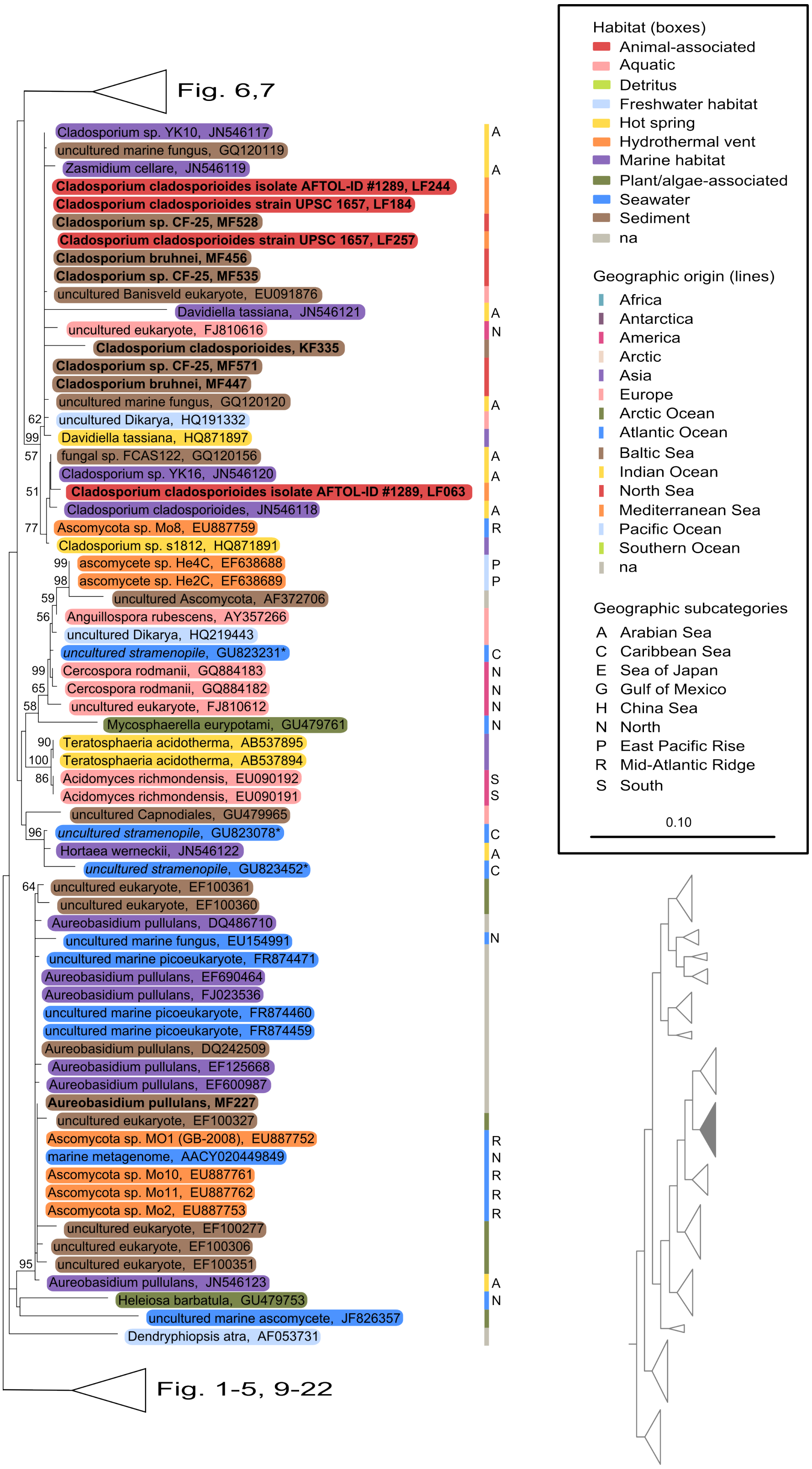

Figure 9: **Subsection of *Dothideomycetes***

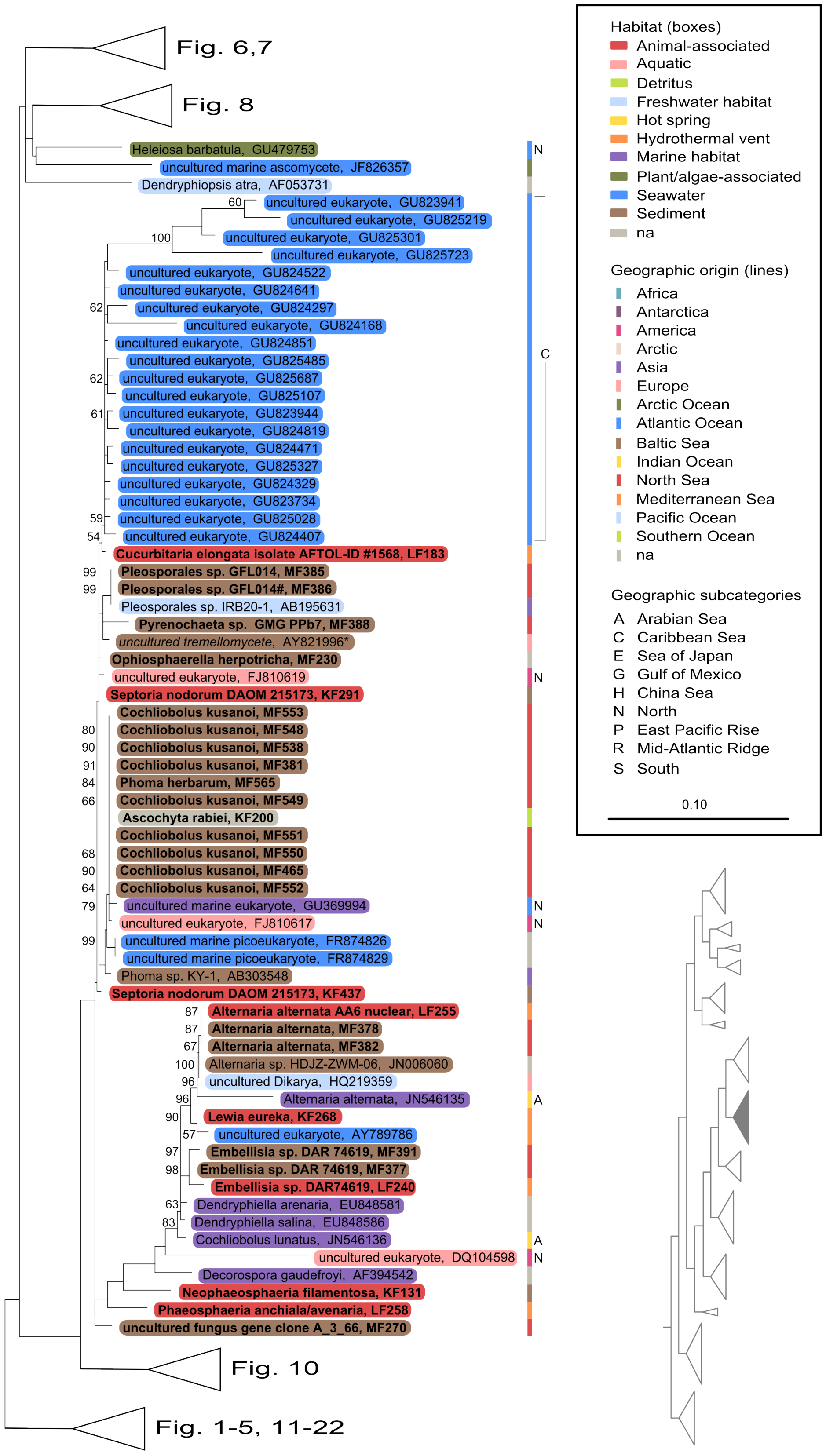

Figure 10: **Subsection of *Dothideomycetes***

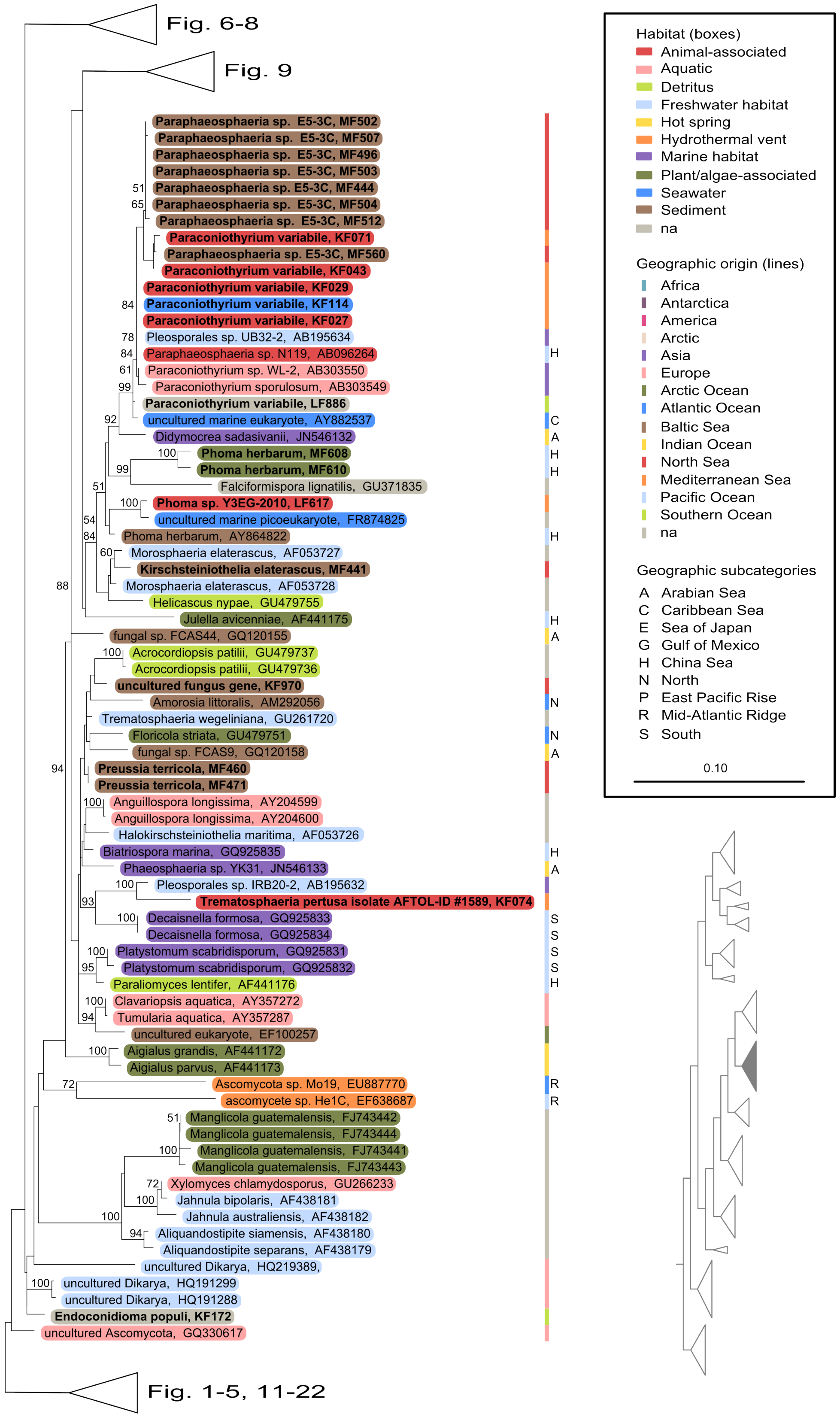

Figure 11: **Subsection of *Leotiomycetes***

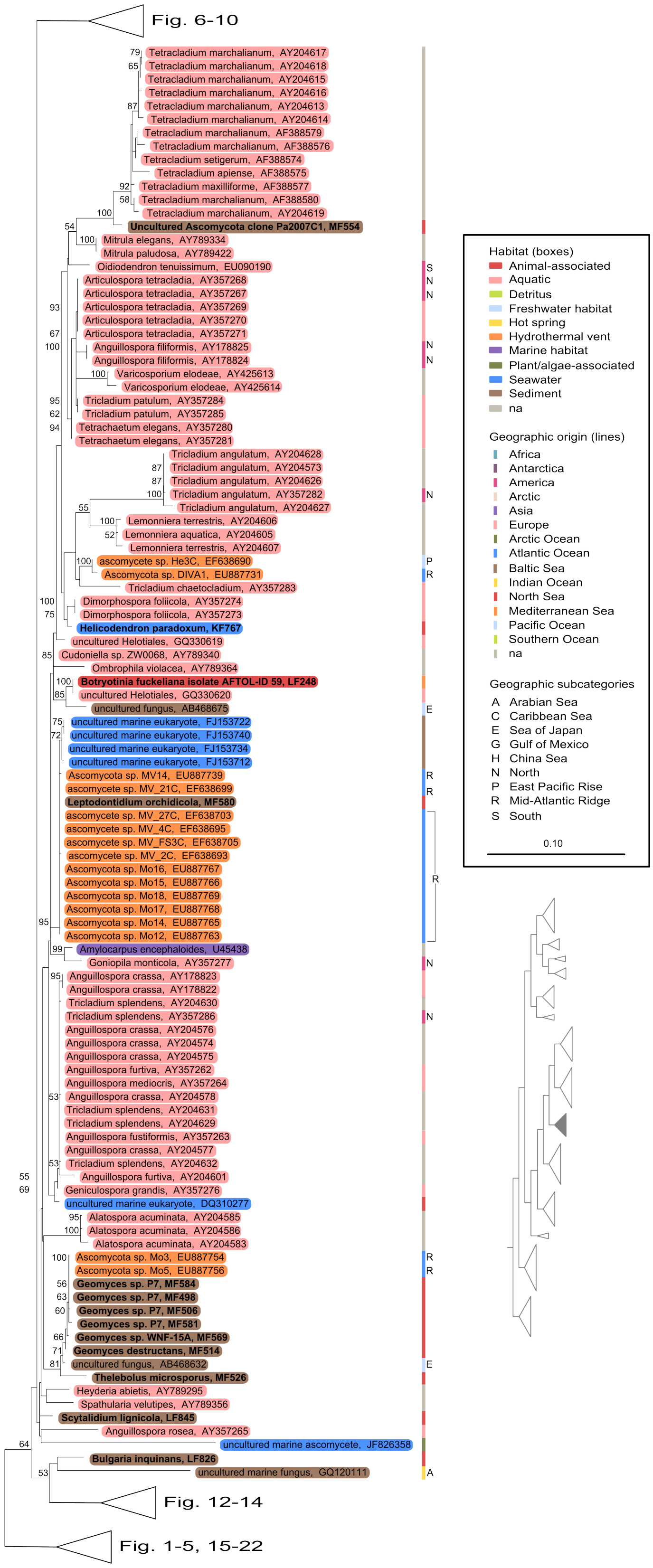

Figure 12: **Subsection of *Sordariomycetes***

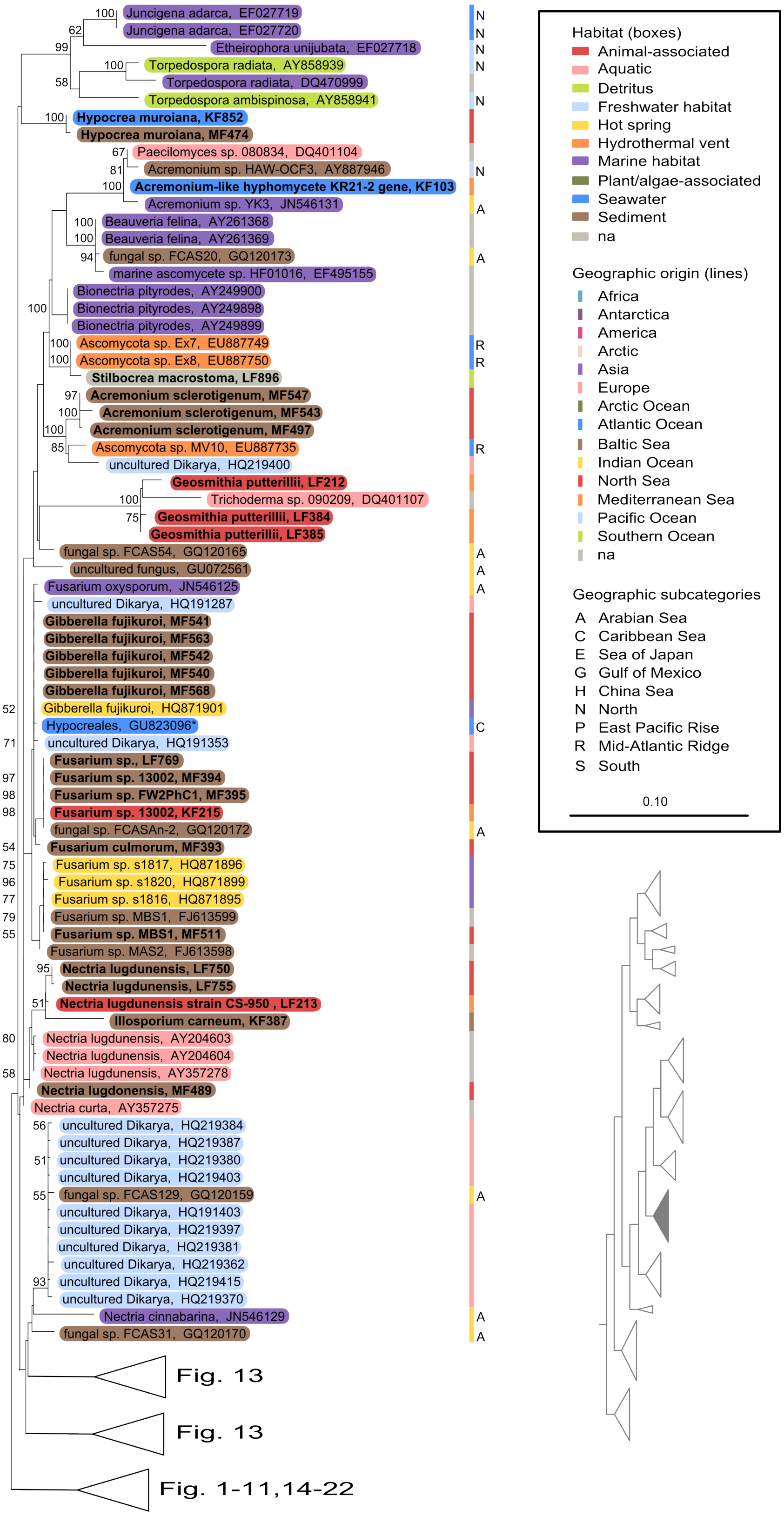

Figure 13: **Subsection of *Sordariomycetes***

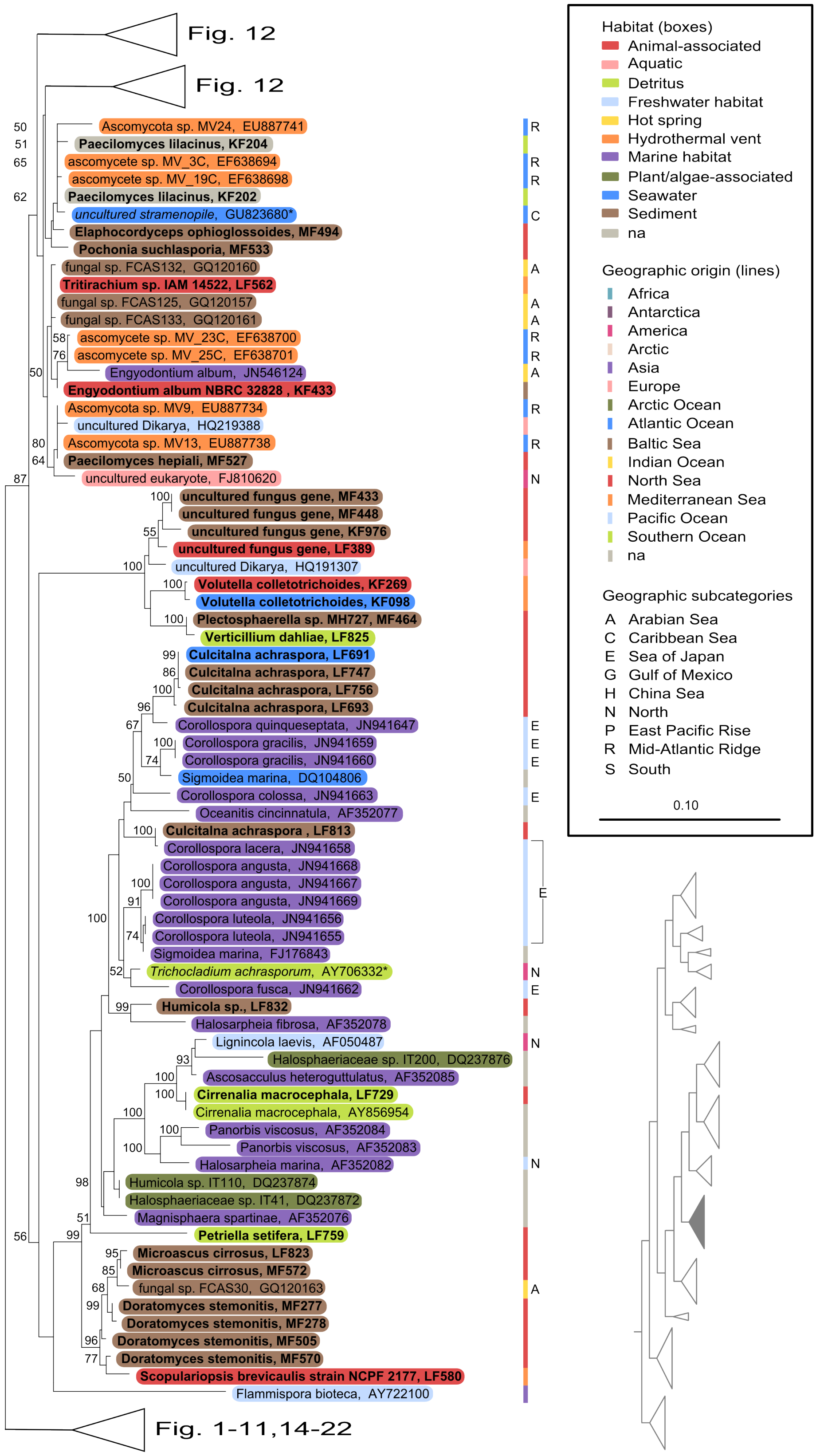

Figure 14: **Subsection of *Sordariomycetes***

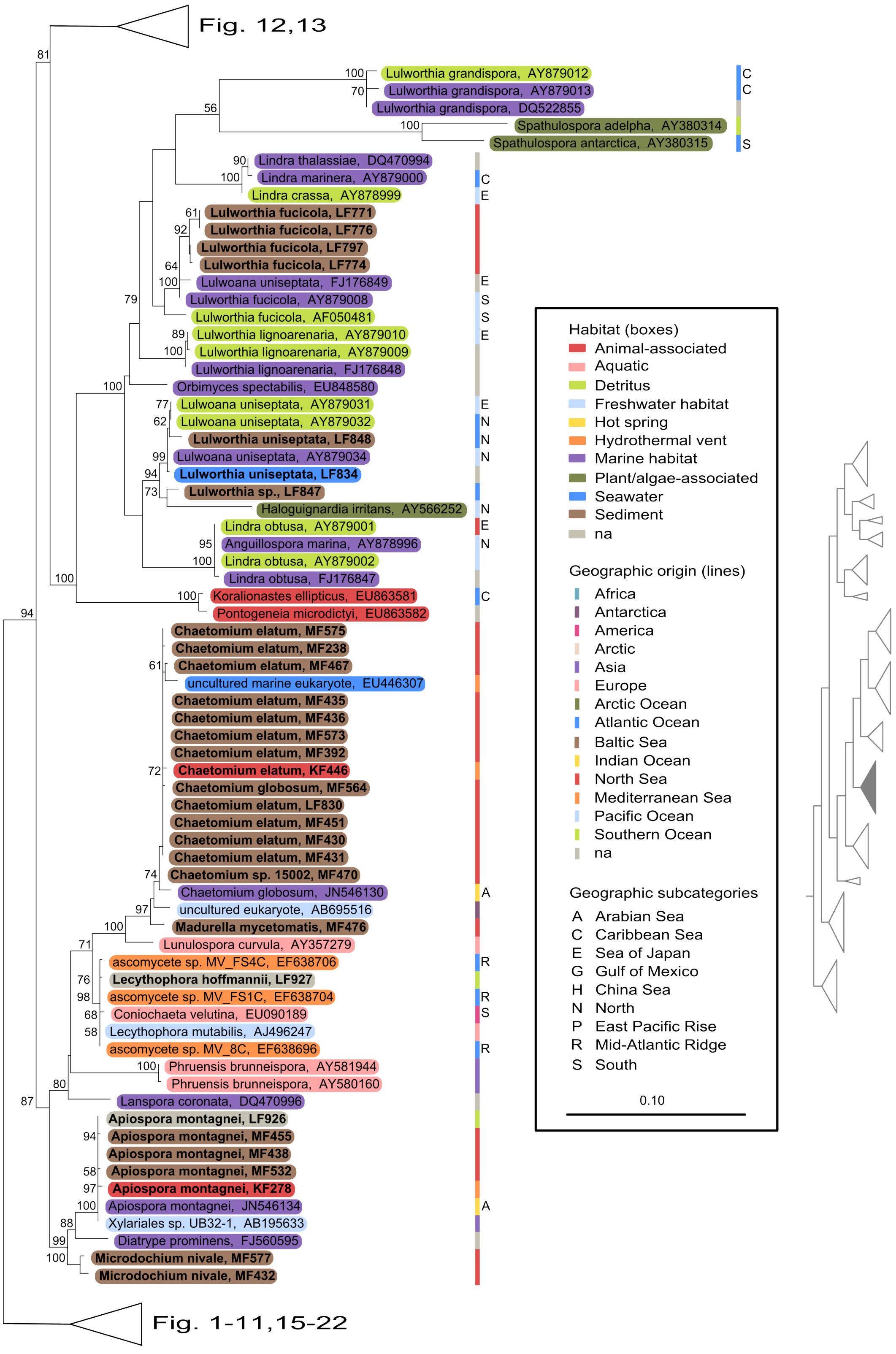

Figure 15: **Subsection of *Saccharomycetes***

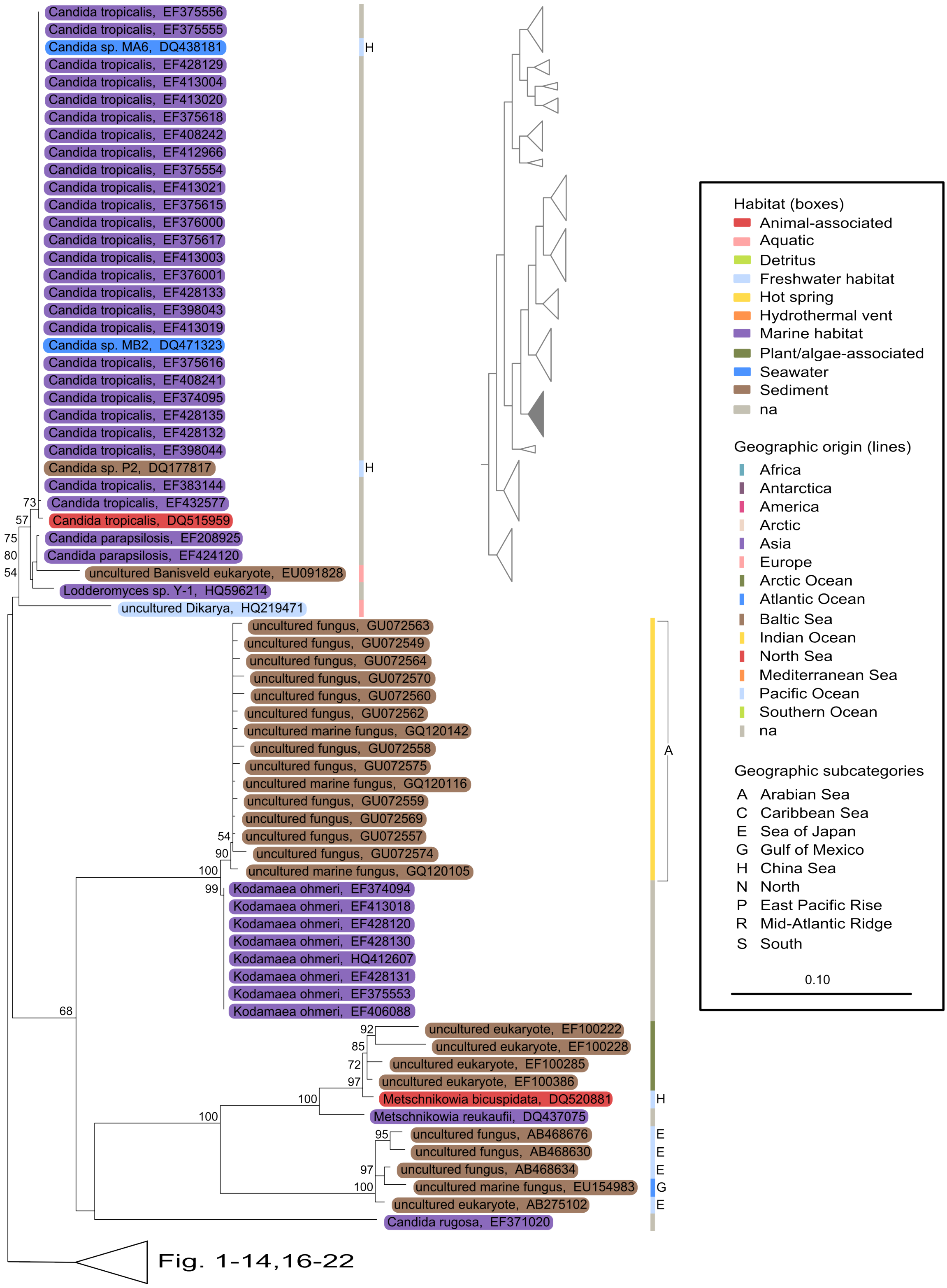

Figure 16: **Subsection of *Saccharomycetes***

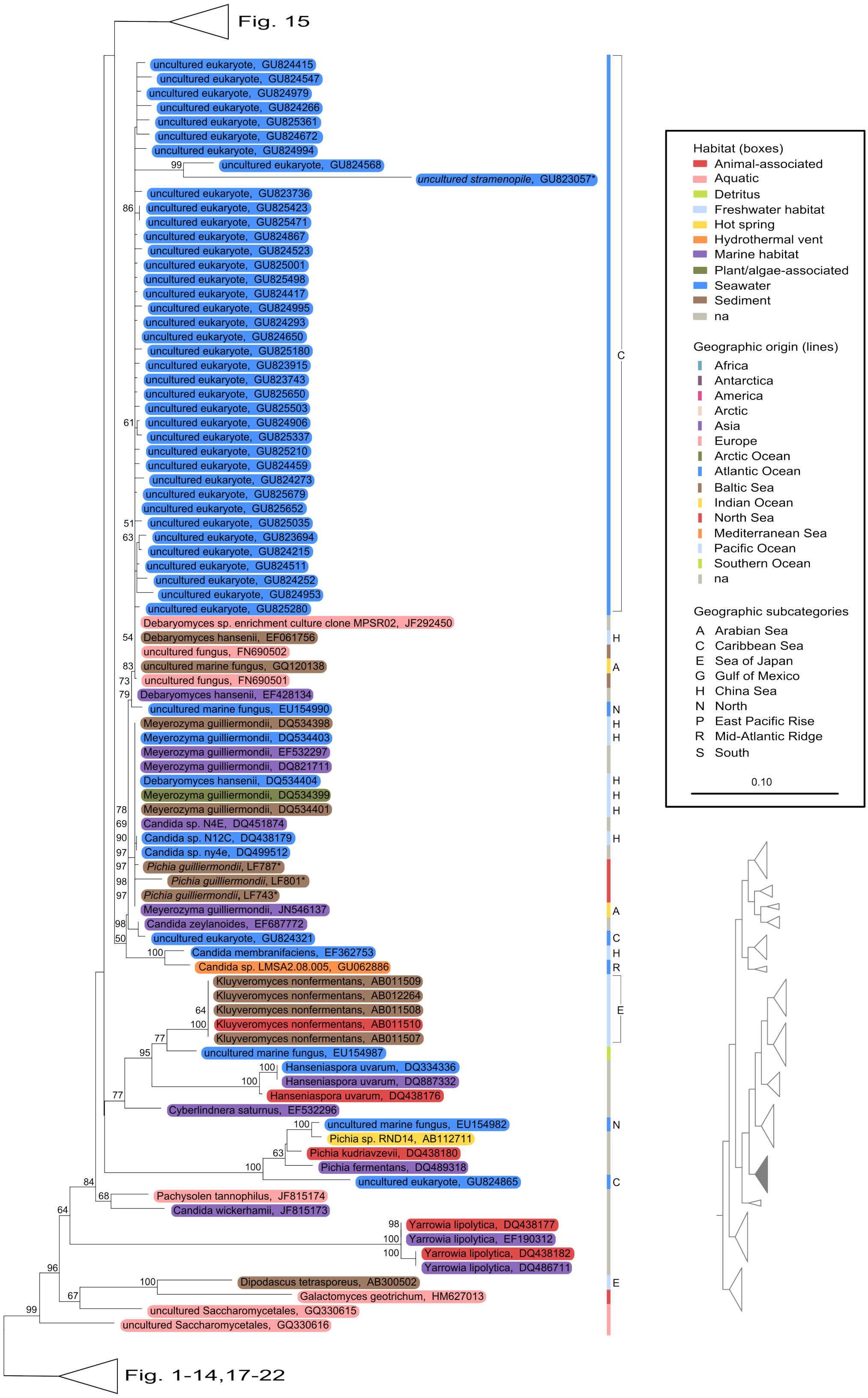

Figure 17: **Subsection of *Taphrinomycotina***

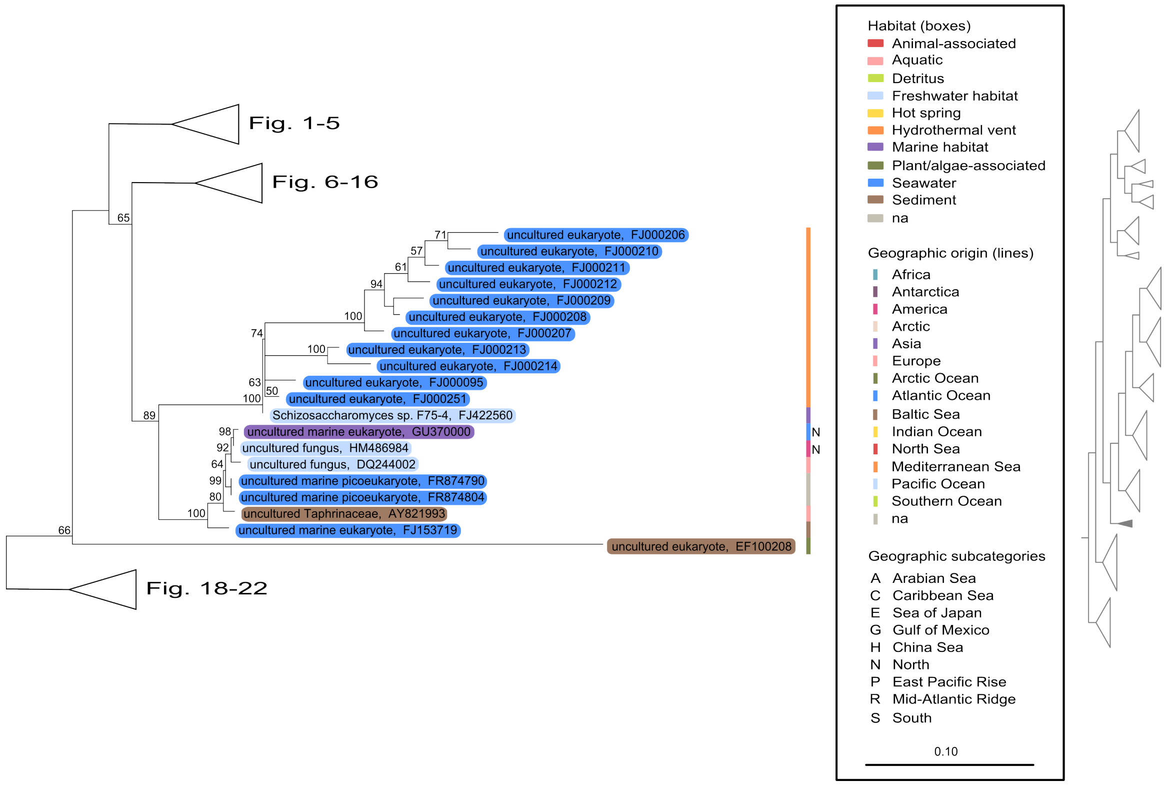

Figure 18: **Subsection of *Chytridiomycota***

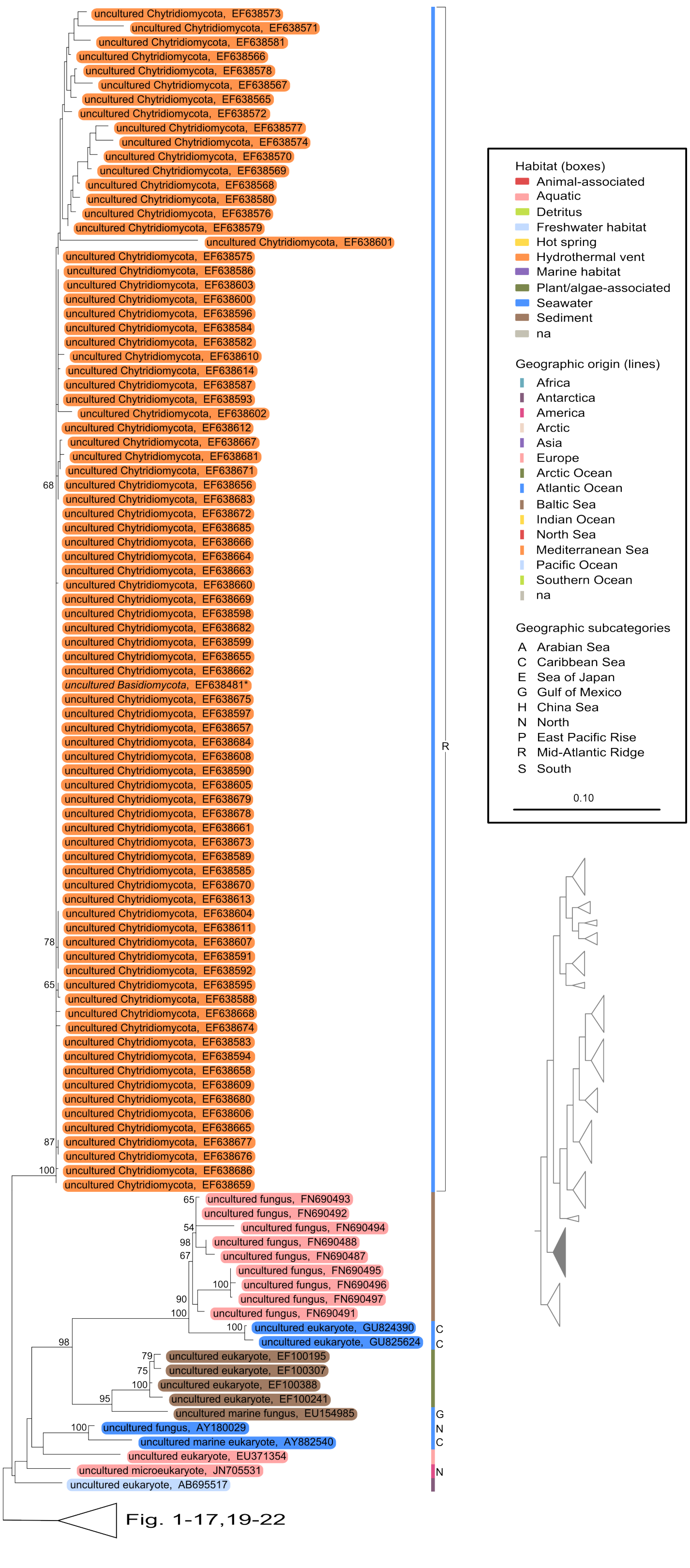

Figure 19: **Subsection of *Chytridiomycota***

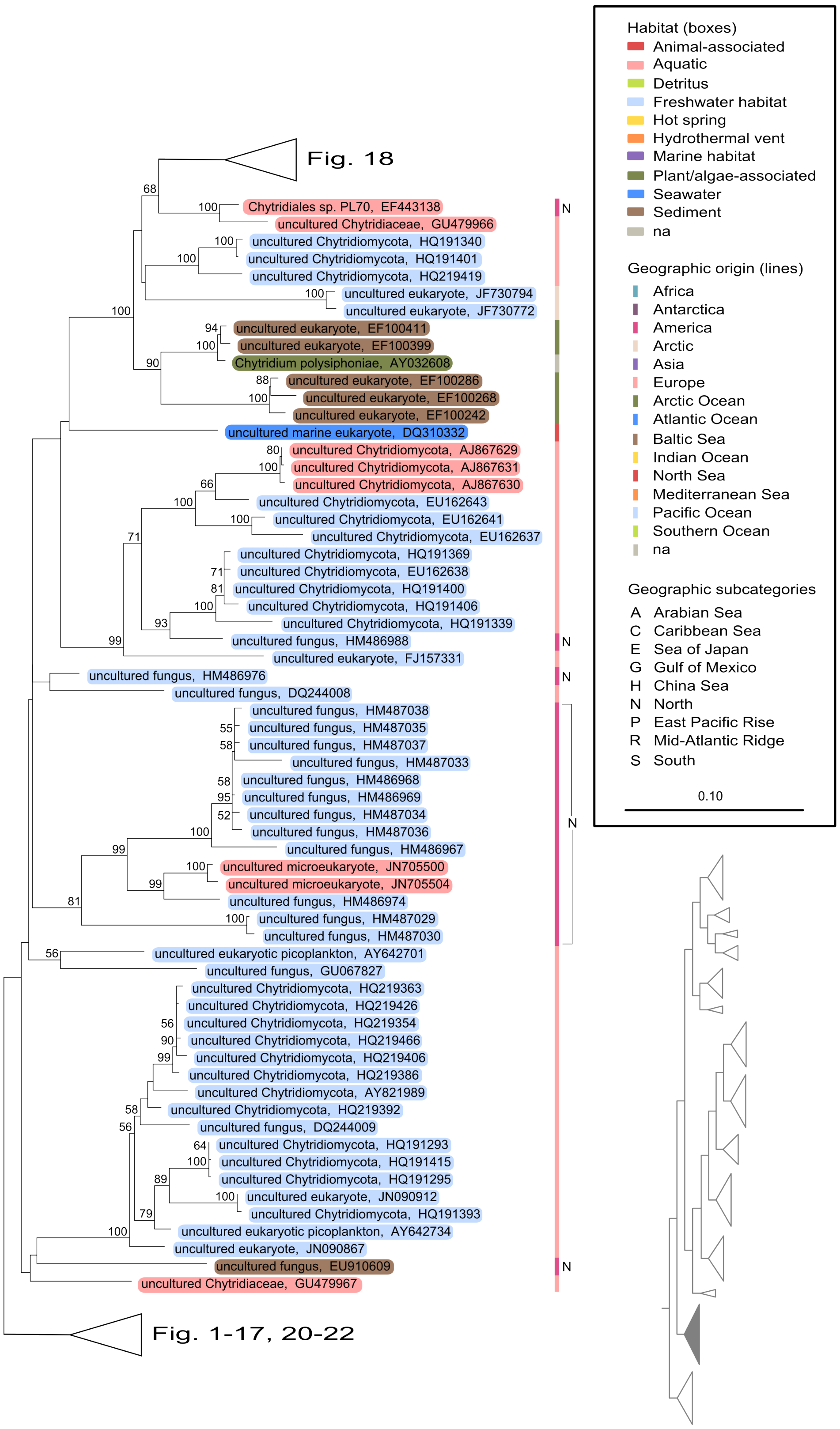

Figure 20: **Subsection of *Chytridiomycota***

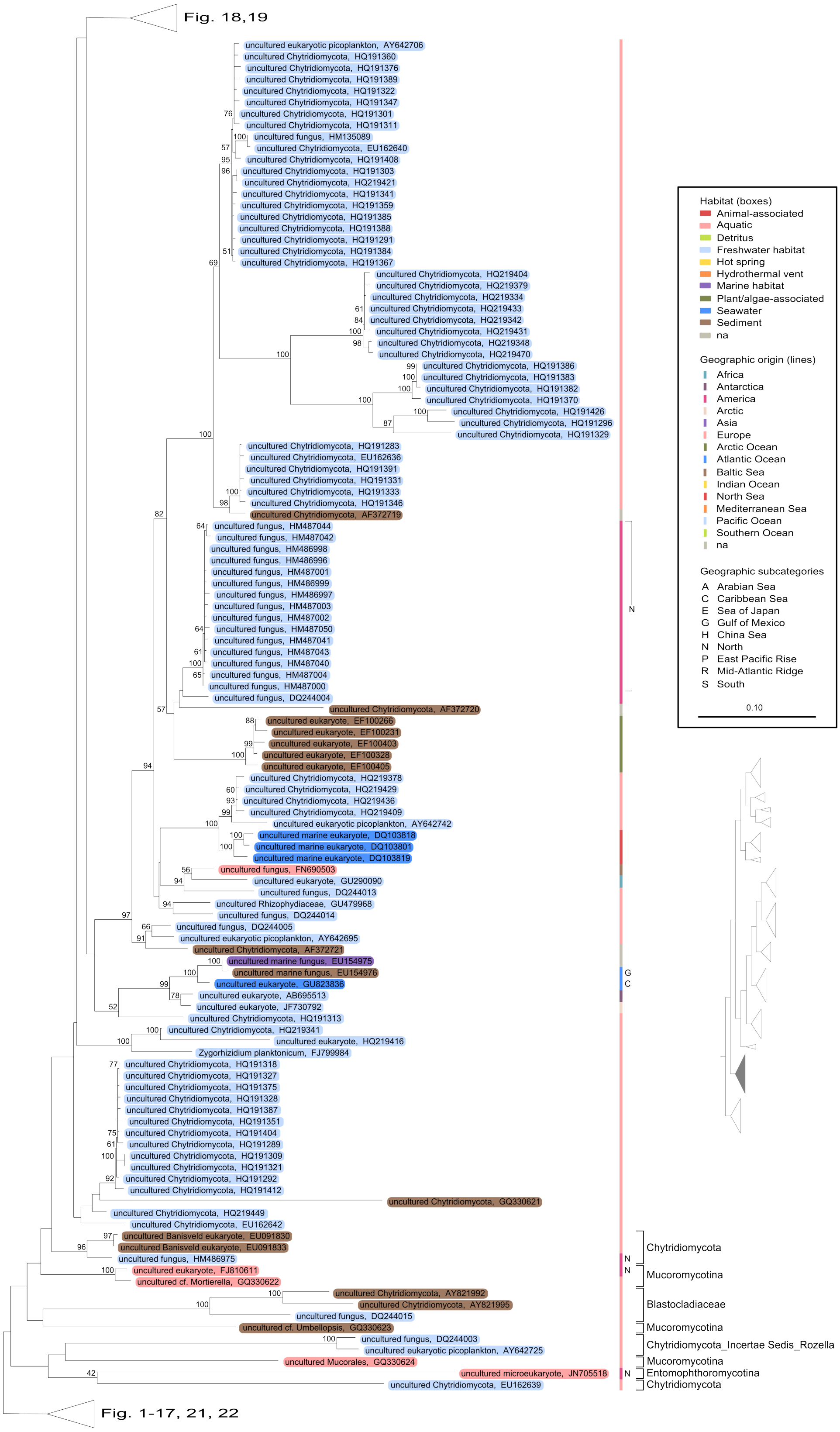

Figure 21: **Subsection of basal fungal lineages**

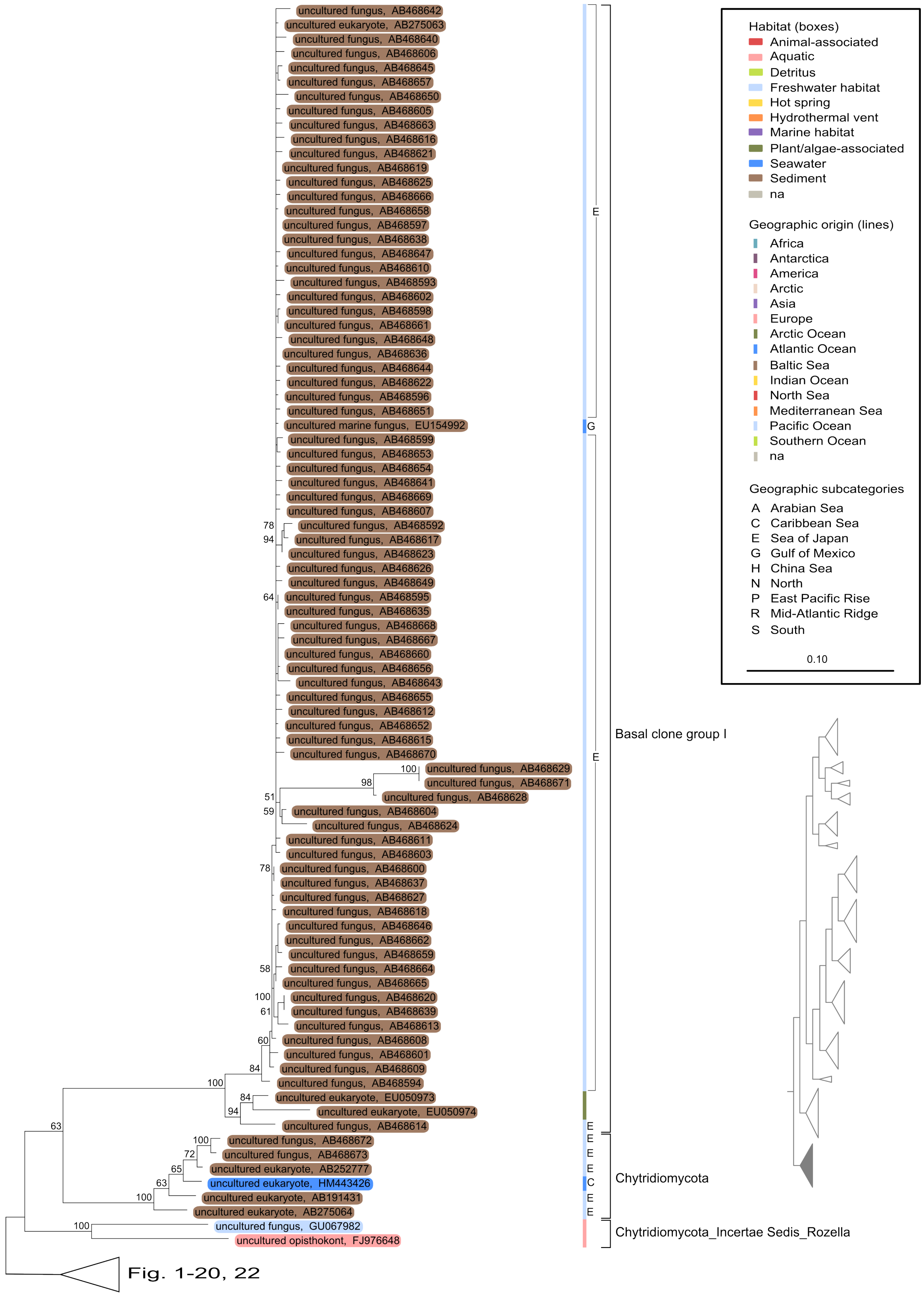

Figure 22: **Subsection of basal fungal lineages**

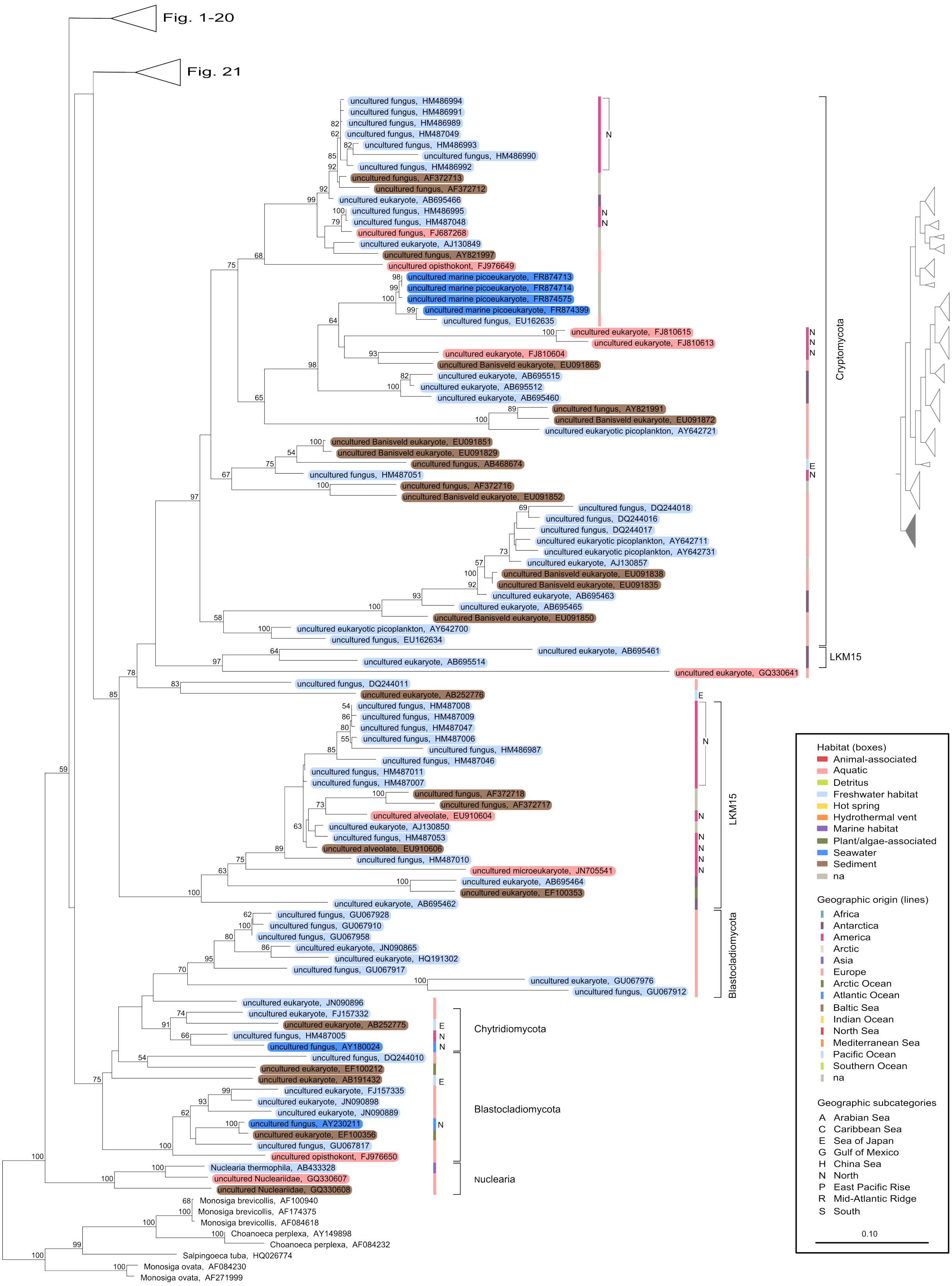

Supplement: S1 Fig — The tree was rooted with sequences of eight species of the Choanoflagellates. Branch support was estimated with 100 bootstrap calculations. Only bootstrap values ≥ 50% are indicated. Sequences derived from the KSMP-Kiel culture collection are printed in bold. Color code, contextual data: colored background of fungal taxa, habitat occurrence of fungal taxa; colored lines beside sequence full name, geographical origin of fungal taxa. Subcategories of the geographical origin displayed as letter to the right of the colored stripe. *, wrong full name annotation of sequence. (PDF) [file pone.0134377.s001.pdf]
