# Supplementary figures and images for: Identification of Habitat-Specific Biomes of Aquatic Fungal Communities Using a Comprehensive Nearly Full-Length 18S rRNA Dataset Enriched with Contextual Data
Source: PLoS One. 2015 Jul 30;10(7):e0134377. doi: 10.1371/journal.pone.0134377 (PMC4520555; doi:10.1371/journal.pone.0134377)

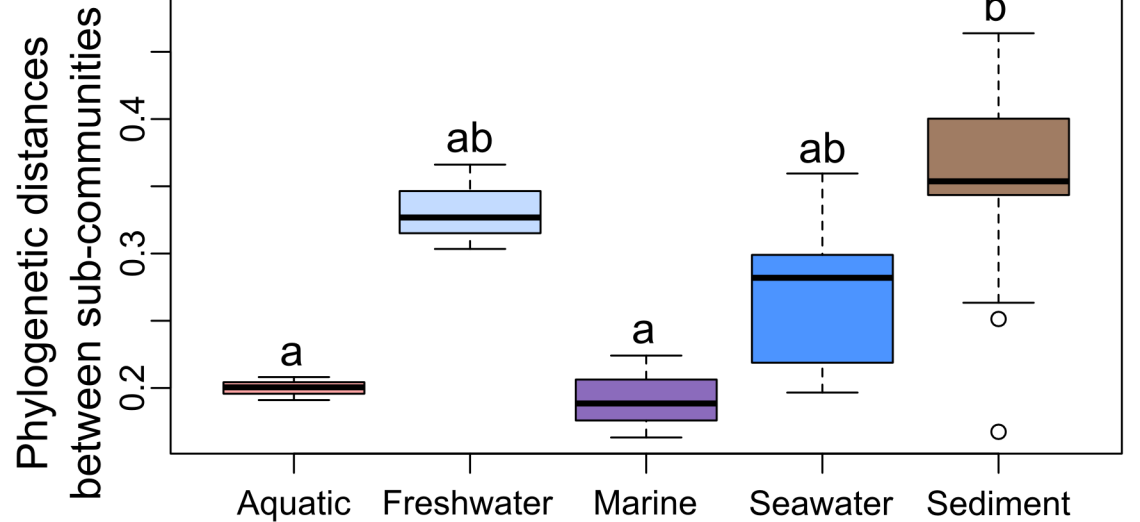

Supplement: S2 Fig — Length of boxes shows variation of phylogenetic distances inferred from pairwise sample comparisons. Black lines, median value. Different letters indicate significant differences (P<0.05) as determined by one-way ANOVA test followed by a Scheffé post hoc test. Number of sub-communities/habitat: four (except sediment: seven). (PDF) [file pone.0134377.s002.pdf]
