# Supplementary material for: Identification of Habitat-Specific Biomes of Aquatic Fungal Communities Using a Comprehensive Nearly Full-Length 18S rRNA Dataset Enriched with Contextual Data
Source: PLoS One. 2015 Jul 30;10(7):e0134377. doi: 10.1371/journal.pone.0134377 (PMC4520555; doi:10.1371/journal.pone.0134377)
Supplement: S1 Table — After inspecting all BLASTN hits, sequences were assigned to a submission name. (PDF) [file pone.0134377.s003.pdf]

Supplementary Table S1: Information on sequences of the KSMP-Kiel culture collection used for this study. After manually inspection of all BLAST-N hits, sequences were assigned to a submission name.

Accession No.	Strain	Submission name	Best BLAST-N hit	Habitat	Geographic Origin
KJ939309	MF566	*Exophiala castellanii*	*Exophiala castellanii*	sediment	North Sea
KJ939310	LF927	*Lecythophora hoffmannii*	*Lecythophora hoffmannii*	na	Southern Ocean
KJ939311	MF583	*Phialophora* sp.	*Phialophora* sp. *GHP 1105*	sediment	North Sea
KJ939312	MF464	*Plectosphaerella* sp.	*Plectosphaerella* sp. *MH727*	sediment	North Sea
KJ939313	MF526	*Thelebolus microsporus*	*Thelebolus microsporus*	sediment	North Sea
KM096133	KF027	*Paraconiothyrium variabile*	*Paraconiothyrium variabile*	animal-associated	Mediterranean Sea
KM096134	KF029	*Paraconiothyrium variabile*	*Paraconiothyrium variabile*	animal-associated	Mediterranean Sea
KM096135	KF043	*Paraconiothyrium variabile*	*Paraconiothyrium variabile*	animal-associated	Mediterranean Sea
KM096136	KF071	*Paraconiothyrium variabile*	*Paraconiothyrium variabile*	animal-associated	Mediterranean Sea
KM096137	KF074	*Pleosporales* sp.	*Trematosphaeria pertusa isolate AFTOL-ID #1589*	animal-associated	Mediterranean Sea
KM096138	KF098	*Acremonium* sp.	*Volutella colletotrichoides*	seawater	Mediterranean Sea
KM096139	KF103	*Sarocladium bactrocephalum*	*Acremonium-like hyphomycete KR21-2 gene*	seawater	Mediterranean Sea
KM096140	KF114	*Paraconiothyrium variabile*	*Paraconiothyrium variabile*	seawater	Mediterranean Se
KM096141	KF128	*Penicillium* sp.	*Penicillium* sp. *- GT 308*	animal-associated	Baltic Sea
KM096142	KF131	*Pleosporales* sp.	*Neophaeosphaeria filamentosa*	animal-associated	Baltic Sea
KM096143	KF133	*Penicillium* sp.	*Penicillium* sp. - GT 308	animal-associated	Baltic Sea
KM096144	KF157	*Rhodotorula lysiniphila*	*Rhodotorula lysiniphila*	na	Southern Ocean
KM096145	KF162	*Rhodosporidium diobovatum*	*Rhodosporidium diobovatum*	na	Southern Ocean
KM096146	KF165	*Rhodotorula lysiniphila*	*Rhodotorula lysiniphila*	na	Southern Ocean
KM096147	KF172	*Dothideomycetes* sp.	*Endoconidioma populi*	na	Southern Ocean
KM096148	KF185	*Exophiala* sp.	*Exophiala* sp. FFC-1	na	Southern Ocean
KM096149	KF200	*Pleosporales* sp.	*Ascochyta rabiei*	na	Southern Ocean
KM096150	KF201	*Penicillium* sp.	*Penicillium* sp. GT308	na	Southern Ocean
KM096151	KF202	*Paecilomyces* sp.	*Paecilomyces lilacinus*	na	Southern Ocean
KM096152	KF204	*Paecilomyces* sp.	*Paecilomyces lilacinus*	na	Southern Ocean
KM096153	KF215	*Fusarium* sp.	*Fusarium* sp. 13002	animal-associated	Mediterranean Sea
KM096154	KF221	*Penicillium* sp.	*Penicillium* sp. GT308	seawater	Mediterranean Sea
KM096155	KF266	*Penicillium* sp.	*Eupenicillium javanicum*	animal-associated	Mediterranean Sea
KM096156	KF268	*Alternaria* sp.	*Lewia eureka*	animal-associated	Mediterranean Sea
KM096157	KF269	*Acremonium* sp.	*Volutella colletotrichoides*	animal-associated	Mediterranean Se
KM096158	KF272	*Penicillium* sp.	*Penicillium* sp. GT308	animal-associated	Mediterranean Se
KM096159	KF277	*Penicillium* sp.	*Penicillium* sp. GT308	animal-associated	Mediterranean Sea
KM096160	KF278	*Arthrinium* sp.	*Apiospora montagnei*	animal-associated	Mediterranean Sea
KM096161	KF291	*Phaeosphaeriaceae* sp.	*Septoria nodorum* DAOM 215173	animal-associated	Baltic Sea
KM096162	KF302	*Penicillium* sp.	*Penicillium* sp. GT308	animal-associated	Mediterranean Sea
KM096163	KF311	*Penicillium* sp.	*Penicillium* sp. GT308	animal-associated	Mediterranean Sea
KM096164	KF335	*Cladosporium* sp.	*Cladosporium cladosporioides*	sediment	Baltic Sea
KM096165	KF387	*Hypocreales* sp.	*Illosporium carneum*	sediment	Baltic Sea
KM096166	KF433	*Engyodontium album*	*Engyodontium album* NBRC 32828	animal-associated	Baltic S
KM096167	KF437	*Phaeosphaeriaceae* sp.	*Septoria nodorum* DAOM 215173	animal-associated	Baltic Sea
KM096168	KF446	*Chaetomium elatum*	*Chaetomium elatum*	animal-associated	Mediterranean Sea
KM096169	KF607	*Penicillium* sp.	*Penicillium* sp. GT308	seawater	North Sea
KM096170	KF612	*Penicillium* sp.	*Penicillium chrysogenum*	seawater	North Sea
KM096171	KF620	*Penicillium* sp.	*Penicillium camemberti*	seawater	North Sea
KM096172	KF648	*Penicillium* sp.	*Eupenicillium javanicum*	seawater	North Sea
KM096173	KF767	*Helicodendron paradoxum*	*Helicodendron paradoxum*	seawater	North Sea
KM096174	KF852	*Trichoderme* sp.	*Hypocrea muroiana*	seawater	North Sea

KM096175	KF869	*Penicillium* sp.	*Penicillium* sp. CCF3812	seawater	North Sea
KM096176	KF970	*Lindgomycetaceae* sp.	uncultured fungus gene	sediment	North Sea
KM096177	KF976	Plectosphaerellaceae sp.	uncultured fungus gene	sediment	North Sea
KM096178	LF063	*Cladosporium* sp.	*Cladosporium cladosporioides isolate AFTOL-ID #1289*	animal-associated	Mediterranean Sea
KM096179	LFM183	*Pleosporales* sp.	*Cucurbitaria elongata* isolate AFTOL-ID #1568	animal-associated	Mediterranean Sea
KM096180	LF184	*Cladosporium* sp.	*Cladosporium cladosporioides strain UPSC 1657*	animal-associated	Mediterranean Sea
KM096181	LF212	*Geosmithia putterillii*	*Geosmithia putterillii*	animal-associated	Mediterranean Se
KM096182	LF213	*Hypocreales* sp.	*Nectria lugdunensis* strain CS-950	animal-associated	Mediterranean Sea
KM096183	LF240	*Pleosporaceae* sp.	*Embellisia* sp. DAR74619	animal-associated	Mediterranean Se
KM096184	LF244	*Cladosporium* sp.	*Cladosporium cladosporioides isolate AFTOL-ID #1289*	animal-associated	Mediterranean Se
KM096185	LF248	*Sclerotiniaceae* sp.	*Botryotinia fuckeliana isolate AFTOL-ID 59*	animal-associated	Mediterranean Sea
KM096186	LF255	*Alternaria* sp.	*Alternaria alternata* AA6 nuclear	animal-associated	Mediterranean Sea
KM096187	LF257	*Cladosporium* sp.	*Cladosporium cladosporioides strain UPSC 1657*	animal-associated	Mediterranean Sea
KM096188	LF258	*Phaeosphaeriaceae* sp.	*Phaeosphaeria anchiala/avenaria*	animal-associated	Mediterranean Sea
KM096189	LF384	*Geosmithia putterillii*	*Geosmithia putterillii*	animal-associated	Mediterranean Se
KM096190	LF385	*Geosmithia putterillii*	*Geosmithia putterillii*	animal-associated	Mediterranean Sea
KM096191	LF387	*Aspergillus versicolor*	*Aspergillus versicolor*	animal-associated	Mediterranean Sea
KM096192	LF389	*Acremonium antarcticum*	uncultured fungus gene	animal-associated	Mediterranean Sea
KM096193	LF562	*Engyodontium* sp.	*Tritirachium* sp. IAM 14522	animal-associated	Mediterranean Sea
KM096194	LF580	*Scopulariopsis brevicaulis*	*Scopulariopsis brevicaulis strain NCPF 2177*	animal-associated	Mediterranean Sea
KM096195	LF617	*Phoma* sp.	*Phoma* sp. Y3EG-2010	animal-associated	Mediterranean Sea
KM096196	LF691	*Corollospora* sp.	*Culcitalna achraspora*	seawater	North Sea
KM096197	LF693	*Corollospora* sp.	*Culcitalna achraspora*	sediment	North Sea
KM096198	LF729	*Cirrenalia macrocephala*	*Cirrenalia macrocephala*	detritus	North Sea
KM096199	LF743	*Debaryomycetaceae* sp.	*Pichia guilliermondii*	sediment	North Sea
KM096200	LF747	*Corollospora quinqueseptata*	*Culcitalna achraspora*	sediment	North Sea
KM096201	LF750	*Hypocreales* sp.	*Nectria lugdunensis*	sediment	North Sea
KM096202	LF755	*Hypocreales* sp.	*Nectria lugdunensis*	sediment	North Sea
KM096203	LF756	*Corollospora* sp.	*Culcitalna achraspora*	sediment	North Sea
KM096204	LF759	*Microascaceae* sp.	*Petriella setifera*	detritus	North Sea
KM096205	LF769	*Fusarium* sp.	*Fusarium* sp.	sediment	North Sea
KM096206	LF771	*Lulworthia fucicola*	*Lulworthia fucicola*	sediment	North Sea
KM096207	LF774	*Lulworthia fucicola*	*Lulworthia fucicola*	sediment	North Sea
KM096208	LF776	*Lulworthia fucicola*	*Lulworthia fucicola*	sediment	North Sea
KM096209	LF787	*Saccharomycetales* sp.	*Pichia guilliermondii*	sediment	North Sea
KM096210	LF797	*Lulworthia fucicola*	*Lulworthia fucicola*	sediment	North Sea
KM096211	LF801	*Saccharomycetales* sp.	*Pichia guilliermondii*	sediment	North Sea
KM096212	LF813	*Corollospora lacera*	*Culcitalna achraspora*	sediment	North Sea
KM096213	LF823	*Microascus cirrosus*	*Microascus cirrosus*	sediment	North Sea
KM096214	LF825	*Plectosphaerellaceae* sp.	*Verticillium dahliae*	detritus	North Sea
KM096215	LF826	*Leotiomycetes* sp.	*Bulgaria inquinans*	sediment	North Sea
KM096216	LF830	*Chaetomium elatum*	*Chaetomium elatum*	sediment	North Sea
KM096217	LF832	*Microascales* sp.	*Humicola* sp.	sediment	North Sea
KM096218	LF834	*Lulworthia uniseptata*	*Lulworthia uniseptata*	seawater	na
KM096219	LF845	*Scytalidium lignicola*	*Scytalidium lignicola*	sediment	North Sea
KM096220	LF847	*Lulworthia* cf. purpurea	*Lulworthia* sp.	sediment	North Atlantic Ocean
KM096221	LF848	*Lulworthia uniseptata*	*Lulworthia uniseptata*	sediment	North Atlantic Ocean
KM096222	LF853	*Penicillium* sp.	*Penicillium* sp.	na	Southern Ocean
KM096223	LF886	*Pleosporales* sp.	*Paraconiothyrium variabile*	na	Southern Ocean
KM096224	LF896	*Hypocreales* sp.	*Stilbocrea macrostoma*	na	Southern Ocean
KM096225	LF926	*Arthrinium arundinis*	*Apiospora montagnei*	na	Southern Ocean
KM096226	LF928	*Penicillium* sp.	*Penicillium* sp. GT308	na	Southern Ocean

KM096227	LF980	*Rhodotorula lysiniphila*	*Rhodotorula lysiniphila*	na	Southern Ocean
KM096228	MF227	*Aureobasidium pullulans*	*Aureobasidium pullulans*	sediment	na
KM096229	MF228	*Tilletiopsis albescens*	*Tilletiopsis albescens*	seawater	na
KM096230	MF229	*Aspergillus versicolor*	*Aspergillus versicolor*	sediment	na
KM096231	MF230	*Ophiosphaerella herpotricha*	*Ophiosphaerella herpotricha*	sediment	na
KM096232	MF238	*Chaetomium elatum*	*Chaetomium elatum*	sediment	North Sea
KM096233	MF270	*Phaeosphaeria* sp.	uncultured fungus gene clone A_3_66	sediment	North Sea
KM096234	MF277	*Doratomyces stemonitis*	*Doratomyces stemonitis*	sediment	North Sea
KM096235	MF278	*Doratomyces stemonitis*	*Doratomyces stemonitis*	sediment	North Sea
KM096236	MF376	*Penicillium* sp.	*Penicillium* sp. 12-02	sediment	North Sea
KM096237	MF377	*Pleosporaceae* sp.	*Embellisia* sp. DAR 74619	sediment	North Sea
KM096238	MF378	*Alternaria* sp.	*Alternaria alternata*	sediment	North Sea
KM096239	MF381	*Epicoccum nigrum*	*Cochliobolus kusanoi*	sediment	North Sea
KM096240	MF382	*Alternaria* sp.	*Alternaria alternata*	sediment	North Sea
KM096241	MF385	*Pleosporales* sp.	*Pleosporales* sp. GFL014	sediment	North Sea
KM096242	MF386	*Pleosporales* sp.	*Pleosporales* sp. GFL014#	sediment	North Sea
KM096243	MF388	*Pyrenochaeta* sp.	*Pyrenochaeta* sp. GMG PPb7	sediment	North Sea
KM096244	MF391	*Embellisia* sp.	*Embellisia* sp. DAR 74619	sediment	North Sea
KM096245	MF392	*Chaetomium elatum*	*Chaetomium elatum*	sediment	North Sea
KM096246	MF393	*Fusarium culmorum*	*Fusarium culmorum*	sediment	North Sea
KM096247	MF394	*Fusarium* sp.	*Fusarium* sp. 13002	sediment	North Sea
KM096248	MF395	*Fusarium* sp.	*Fusarium* sp. FW2PhC1	sediment	North Sea
KM096249	MF396	*Penicillium* citrinum	*Penicillium* sp. CTCC 480032	animal-associated	South Pacific Ocean
KM096250	MF399	*Penicillium* citrinum	*Penicillium* sp. CTCC 480032	animal-associated	South Pacific Ocean
KM096251	MF408	*Penicillium* citrinum	*Penicillium* sp. CTCC 480032	animal-associated	South Pacific Ocean
KM096252	MF410	*Penicillium* citrinum	*Penicillium* sp. CTCC 480032	animal-associated	South Pacific Ocean
KM096253	MF419	*Byssochlamys spectsbilis*	*Paecilomyces variotii*	animal-associated	South Pacific Ocean
KM096254	MF423	*Byssochlamys spectsbilis*	*Paecilomyces varioti*i	animal-associated	South Pacific Ocean
KM096255	MF424	*Byssochlamys spectsbilis*	*Paecilomyces variotii*	animal-associated	South Pacific Ocean
KM096256	MF430	*Chaetomium elatum*	*Chaetomium elatum*	sediment	North Sea
KM096257	MF431	*Chaetomium elatum*	*Chaetomium elatum*	sediment	North Sea
KM096258	MF432	*Microdochium nivale*	*Microdochium nivale*	sediment	North Sea
KM096259	MF433	*Plectosphaerellaceae* sp.	uncultured fungus gene	sediment	North Sea
KM096260	MF435	*Chaetomium elatum*	*Chaetomium elatum*	sediment	North Sea
KM096261	MF436	*Chaetomium elatum*	*Chaetomium elatum*	sediment	North Sea
KM096262	MF438	*Arthrinium* sp.	*Apiospora montagnei*	sediment	North Sea
KM096263	MF440	*Penicillium namyslowskii*	*Penicillium namyslowskii*	sediment	North Sea
KM096264	MF441	*Kirschsteiniothelia elaterascus*	*Kirschsteiniothelia elaterascus*	sediment	North Sea
KM096265	MF444	*Pleosporales* sp.	*Paraphaeosphaeria* sp. E5-3C	sediment	North Sea
KM096266	MF445	*Penicillium* sp.	*Penicillium* sp. GT308	sediment	North Sea
KM096267	MF446	*Penicillium* sp.	*Penicillium expansum*	sediment	North Sea
KM096268	MF447	*Cladosporium* sp.	*Cladosporium bruhnei*	sediment	North Sea
KM096269	MF448	*Plectosphaerellaceae* sp.	uncultured fungus gene	sediment	North Sea
KM096270	MF449	*Penicillium* sp.	*Penicillium camemberti*	sediment	North Sea
KM096271	MF450	*Penicillium* sp.	*Penicillium chrysogenum*	sediment	North Sea
KM096272	MF451	*Chaetomium elatum*	*Chaetomium elatum*	sediment	North Sea
KM096273	MF452	*Penicillium* sp.	*Penicillium expansum*	sediment	North Sea
KM096274	MF455	*Arthrinium* sp.	*Apiospora montagnei*	sediment	North Sea
KM096275	MF456	*Cladosporium* sp.	*Cladosporium bruhnei*	sediment	North Sea
KM096276	MF459	*Penicillium* sp.	*Penicillium* sp. GT308	sediment	North Sea
KM096277	MF460	*Preussia* sp.	*Preussia terricola*	sediment	North Sea
KM096278	MF461	*Penicillium* sp.	*Penicillium* sp. GT308	sediment	North Sea

KM096279	MF462	*Penicillium* sp.	*Penicillium chrysogenum*	sediment	North Sea
KM096280	MF463	*Penicillium* sp.	*Penicillium* sp. 1F	sediment	North Sea
KM096281	MF465	*Pleosporales* sp.	*Cochliobolus kusanoi*	sediment	North Sea
KM096282	MF466	*Penicillium* sp.	*Penicillium chrysogenum*	sediment	North Sea
KM096283	MF467	*Chaetomium elatum*	*Chaetomium elatum*	sediment	North Sea
KM096284	MF468	*Penicillium* sp.	*Penicillium* sp. GT308	sediment	North Sea
KM096285	MF470	*Chaetomium* sp.	*Chaetomium* sp. 15002	sediment	North Sea
KM096286	MF471	*Preussia sp.*	*Preussia terricola*	sediment	North Sea
KM096287	MF472	*Penicillium* sp.	*Penicillium* sp. GT308	sediment	North Sea
KM096288	MF474	*Hypocreaceae* sp.	*Hypocrea muroiana*	sediment	North Sea
KM096289	MF475	*Penicillium* sp.	*Penicillium* sp. GT308	sediment	North Sea
KM096290	MF476	*Sordariales* sp.	*Madurella mycetomatis*	sediment	North Sea
KM096291	MF477	*Penicillium* sp.	*Penicillium* sp. GT308	sediment	North Sea
KM096292	MF478	*Penicillium* sp.	*Penicillium* sp. GT308	sediment	North Sea
KM096293	MF479	*Penicillium* sp.	*Penicillium expansum*	sediment	North Sea
KM096294	MF482	*Penicillium* sp.	*Penicillium expansum*	sediment	North Sea
KM096295	MF484	*Penicillium* sp.	*Penicillium chrysogenum*	sediment	North Sea
KM096296	MF485	*Penicillium* sp.	*Penicillium chrysogenum*	sediment	North Sea
KM096297	MF486	*Penicillium* sp.	*Penicillium expansum*	sediment	North Sea
KM096298	MF487	*Penicillium* sp.	*Penicillium chrysogenum*	sediment	North Sea
KM096299	MF488	*Penicillium* sp.	*Penicillium expansum*	sediment	North Sea
KM096300	MF489	*Hypocreales* sp.	*Nectria lugdonensis*	sediment	North Sea
KM096301	MF490	*Penicillium* sp.	*Penicillium camemberti*	sediment	North Sea
KM096302	MF492	*Penicillium* sp.	*Penicillium expansum*	sediment	North Sea
KM096303	MF493	*Penicillium* sp.	*Penicillium chrysogenum*	sediment	North Sea
KM096304	MF494	*Hypocreales* sp.	*Elaphocordyceps ophioglossoides*	sediment	North Sea
KM096305	MF495	*Penicillium* sp.	*Penicillium expansum*	sediment	North Sea
KM096306	MF496	*Pleosporales* sp.	*Paraphaeosphaeria* sp. E5-3C	sediment	North Sea
KM096307	MF497	*Acremonium sclerotigenum*	*Acremonium sclerotigenum*	sediment	North Sea
KM096308	MF498	*Geomyces* sp.	*Geomyces* sp. P7	sediment	North Sea
KM096309	MF502	*Pleosporales* sp.	*Paraphaeosphaeria* sp. E5-3C	sediment	North Sea
KM096310	MF503	*Pleosporales* sp.	*Paraphaeosphaeria* sp. E5-3C	sediment	North Sea
KM096311	MF504	*Pleosporales* sp.	*Paraphaeosphaeria* sp. E5-3C	sediment	North Sea
KM096312	MF505	*Doratomyces stemonitis*	*Doratomyces stemonitis*	sediment	North Sea
KM096313	MF506	*Geomyces* sp.	*Geomyces* sp. P7	sediment	North Sea
KM096314	MF507	*Pleosporales* sp.	*Paraphaeosphaeria* sp. E5-3C	sediment	North Sea
KM096315	MF508	*Penicillium* sp.	*Penicillium tardum*	sediment	North Sea
KM096316	MF509	*Penicillium* sp.	*Penicillium brevicompactum*	sediment	North Sea
KM096317	MF510	*Penicillium* sp.	*Penicillium* sp. GT308	sediment	North Sea
KM096318	MF511	*Fusarium* sp.	*Fusarium* sp. MBS1	sediment	North Sea
KM096319	MF512	*Pleosporales* sp.	*Paraphaeosphaeria* sp. E5-3C	sediment	North Sea
KM096320	MF513	*Penicillium* sp.	*Eupenicillium javanicum*	sediment	North Sea
KM096321	MF514	*Geomyces* sp.	*Geomyces destructans*	sediment	North Sea
KM096322	MF515	*Penicillium* sp.	*Penicillium expansum*	sediment	North Sea
KM096323	MF518	*Penicillium* sp.	*Eupenicillium javanicum*	sediment	North Sea
KM096324	MF520	*Penicillium* sp.	*Penicillium* sp. 1F	sediment	North Sea
KM096325	MF521	*Penicillium* sp.	*Penicillium chrysogenum*	sediment	North Sea
KM096326	MF522	*Penicillium namyslowskii*	*Penicillium namyslowskii*	sediment	North Sea
KM096327	MF523	*Penicillium* sp.	*Penicillium* sp. GT308	sediment	North Sea
KM096328	MF524	*Penicillium* sp.	*Penicillium camemberti*	sediment	North Sea
KM096329	MF525	*Penicillium* sp.	*Penicillium* sp. GT-308	sediment	North Sea
KM096330	MF527	*Hypocreales* sp.	*Paecilomyces hepiali*	sediment	North Sea

KM096331	MF528	*Cladosporium* sp.	*Cladosporium* sp. CF-25	sediment	North Sea
KM096332	MF530	*Penicillium* sp.	*Penicillium chrysogenum*	sediment	North Sea
KM096333	MF532	*Arthrinium* sp.	*Apiospora montagnei*	sediment	North Sea
KM096334	MF533	*Pochonia suchlasporia*	*Pochonia suchlasporia*	sediment	North Sea
KM096335	MF534	*Eurotium* sp.	*Eurotium herbariorum*	sediment	North Sea
KM096336	MF535	*Cladosporium* sp.	*Cladosporium* sp. CF-25	sediment	North Sea
KM096337	MF538	*Pleosporales* sp.	*Cochliobolus kusanoi*	sediment	North Sea
KM096338	MF540	*Nectriaceae* sp.	*Gibberella fujikuroi*	sediment	North Sea
KM096339	MF541	*Nectriaceae* sp.	*Gibberella fujikuroi*	sediment	North Sea
KM096340	MF542	*Nectriaceae* sp.	*Gibberella fujikuroi*	sediment	North Sea
KM096341	MF543	*Acremonium sclerotigenum*	*Acremonium sclerotigenum*	sediment	North Sea
KM096342	MF544	*Aspergillus versicolor*	*Aspergillus versicolor*	sediment	North Sea
KM096343	MF545	*Aspergillus versicolor*	*Aspergillus versicolor*	sediment	North Sea
KM096344	MF546	*Aspergillus versicolor*	*Aspergillus versicolor*	sediment	North Sea
KM096345	MF547	*Acremonium sclerotigenum*	*Acremonium sclerotigenum*	sediment	North Sea
KM096346	MF548	*Pleosporales* sp.	*Cochliobolus kusanoi*	sediment	North Sea
KM096347	MF549	*Pleosporales* sp.	*Cochliobolus kusanoi*	sediment	North Sea
KM096348	MF550	*Pleosporales* sp.	*Cochliobolus kusanoi*	sediment	North Sea
KM096349	MF551	*Pleosporales* sp.	*Cochliobolus kusanoi*	sediment	North Sea
KM096350	MF552	*Pleosporales* sp.	*Cochliobolus kusanoi*	sediment	North Sea
KM096351	MF553	*Pleosporales* sp.	*Cochliobolus kusanoi*	sediment	North Sea
KM096352	MF554	*Tetracladium* sp.	Uncultured Ascomycota clone Pa2007C1	sediment	North Sea
KM096353	MF555	*Penicillum* sp.	*Penicillum tardum*	sediment	North Sea
KM096354	MF557	*Aspergillus versicolor*	*Aspergillus versicolor*	sediment	North Sea
KM096355	MF560	*Pleosporales* sp.	*Paraphaeosphaeria* sp. E5-3C	sediment	North Sea
KM096356	MF563	*Nectriaceae* sp.	*Gibberella fujikuroi*	sediment	North Sea
KM096357	MF564	*Chaetomium globosum*	*Chaetomium globosum*	sediment	North Sea
KM096358	MF565	*Pleosporales* sp.	*Phoma herbarum*	sediment	North Sea
KM096359	MF568	*Nectriaceae* sp.	*Gibberella fujikuroi*	sediment	North Sea
KM096360	MF569	*Geomyces* sp.	*Geomyces* sp. WNF-15A	sediment	North Sea
KM096361	MF570	*Doratomyces stemonitis*	*Doratomyces stemonitis*	sediment	North Sea
KM096362	MF571	*Cladosporium* sp.	*Cladosporium* sp. CF-25	sediment	North Sea
KM096363	MF572	*Microascus cirrosus*	*Microascus cirrosus*	sediment	North Sea
KM096364	MF573	*Chaetomium elatum*	*Chaetomium elatum*	sediment	North Sea
KM096365	MF574	Penicillium sp.	*Eupenicillium javanicum*	sediment	North Sea
KM096366	MF575	*Chaetomium elatum*	*Chaetomium elatum*	sediment	North Sea
KM096367	MF577	*Microdochium nivale*	*Microdochium nivale*	sediment	North Sea
KM096368	MF580	*Helotiales* sp.	*Leptodontidium orchidicola*	sediment	North Sea
KM096369	MF581	*Geomyces* sp.	*Geomyces* sp. P7	sediment	North Sea
KM096370	MF584	*Geomyces* sp.	*Geomyces* sp. P7	sediment	North Sea
KM096371	MF607	*Trichosporon laibachii*	*Trichosporon laibachii*	plant/algae-associated	China Sea
KM096372	MF608	*Pleosporales* sp.	*Phoma herbarum*	plant/algae-associated	China Sea
KM096373	MF609	*Penicillium verruculosum*	*Penicillium purpurogenum*	plant/algae-associated	China Sea
KM096374	MF610	*Pleosporales* sp.	*Phoma herbarum*	plant/algae-associated	China Sea

Abbreviation: No information available, na
