# Supplementary material for: Identification of Habitat-Specific Biomes of Aquatic Fungal Communities Using a Comprehensive Nearly Full-Length 18S rRNA Dataset Enriched with Contextual Data
Source: PLoS One. 2015 Jul 30;10(7):e0134377. doi: 10.1371/journal.pone.0134377 (PMC4520555; doi:10.1371/journal.pone.0134377)
Supplement: S2 Table — Fungal sequences were subjected to BLASTN analysis and checked for their taxonomic placement in the eukaryotic guide-tree of the SILVA release 111. Sequences were classified depending on combined results from the methods mentioned above, as well as literature searches. (PDF) [file pone.0134377.s004.pdf]

Supplementary Table S2: New taxonomic assignment of sequences of basal fungal lineages. Fungal sequences were subjected to BLAST-N analysis and checked for their taxonomic placement in the eukaryotic guide-tree of the SILVA release 111. Sequences were classified depending on combined results from the methods mentioned above as well as literature searches.

Accession number	Name	New classification based on combined results	Clustering of the sequence in the eukaryotic guide tree of SILVA	Best BLAST-N hit Name	Accession number	E.value	Identity
AB191431	Uncultured fungus	*Chytridiomycota*	*Chytridiomycota*	*Basidiobolus haptosporus*	AF113413.1	0.0	91
AB191432	Uncullltured eukaryote	*Blastocladiomycota*	*Blastocladiomycota*	*Rhizophlyctis rosea*	NG_017175.1	0.0	91
AB252775	Uncultured eukaryote	*Chytridiomycota*	*Chytridiomycota*	*Blastocladiales sp.*	EF565163.1	0.0	91
AB252776	Uncultured eukaryote	Fungi	Nucletmycea_Fonticula	*Rhizophydium sp.*	AF164270.2	0.0	87
AB252777	Uncultured eukaryote	*Chytridiomycota*	*Chytridiomycota*	*Basidiobolus haptosporus*	AF113413.1	0.0	91
AB275063	Uncultured fungus	*Chytridiomycota*	*Chytridiomycota*	*Catenomyces sp.*	AY635830.1	0.0	90
AB275064	Uncultured fungus	*Chytridiomycota*	*Chytridiomycota*	*Endogone lactiflua*	DQ536471.1	0.0	91
AB433328	*Nuclearia thermophila*	*Nuclearia*	Nucletmycea_Nuclearia	*Nuclearia thermophila*	AB433328.1	0.0	100
AB468592	Uncultured fungus	Basal clone group I	*Chytridiomycota*	*Physoderma dulichii*	DQ536472.1	0.0	90
AB468593	Uncultured fungus	Basal clone group I	*Chytridiomycota*	*Physoderma dulichii*	DQ536472.1	0.0	91
AB468594	Uncultured fungus	Basal clone group I	*Chytridiomycota*	*Urophlyctis pulposa*	HQ888719.1	0.0	91
AB468595	Uncultured fungus	Basal clone group I	*Chytridiomycota*	*Physoderma dulichii*	DQ536472.1	0.0	91
AB468596	Uncultured fungus	Basal clone group I	*Chytridiomycota*	*Physoderma dulichii*	DQ536472.1	0.0	91
AB468597	Uncultured fungus	Basal clone group I	*Chytridiomycota*	*Physoderma dulichii*	DQ536472.1	0.0	91
AB468598	Uncultured fungus	Basal clone group I	*Chytridiomycota*	*Physoderma dulichii*	DQ536472.1	0.0	91
AB468599	Uncultured fungus	Basal clone group I	*Chytridiomycota*	*Physoderma dulichii*	DQ536472.1	0.0	91
AB468600	Uncultured fungus	Basal clone group I	*Chytridiomycota*	*Physoderma dulichii*	DQ536472.1	0.0	91
AB468601	Uncultured fungus	Basal clone group I	*Chytridiomycota*	*Urophlyctis pulposa*	HQ888719.1	0.0	90
AB468602	Uncultured fungus	Basal clone group I	*Chytridiomycota*	*Physoderma dulichii*	DQ536472.1	0.0	91
AB468603	Uncultured fungus	Basal clone group I	*Chytridiomycota*	*Physoderma dulichii*	DQ536472.1	0.0	91
AB468604	Uncultured fungus	Basal clone group I	*Chytridiomycota*	*Physoderma dulichii*	DQ536472.1	0.0	91
AB468605	Uncultured fungus	Basal clone group I	*Chytridiomycota*	*Physoderma dulichii*	DQ536472.1	0.0	91
AB468606	Uncultured fungus	Basal clone group I	*Chytridiomycota*	*Physoderma dulichii*	DQ536472.1	0.0	91
AB468607	Uncultured fungus	Basal clone group I	*Chytridiomycota*	*Physoderma dulichii*	DQ536472.1	0.0	91
AB468608	Uncultured fungus	Basal clone group I	*Chytridiomycota*	*Physoderma dulichii*	DQ536472.1	0.0	91
AB468609	Uncultured fungus	Basal clone group I	*Chytridiomycota*	*Urophlyctis pulposa*	HQ888719.1	0.0	91
AB468610	Uncultured fungus	Basal clone group I	*Chytridiomycota*	*Physoderma dulichii*	DQ536472.1	0.0	91
AB468611	Uncultured fungus	Basal clone group I	*Chytridiomycota*	*Physoderma dulichii*	DQ536472.1	0.0	90
AB468612	Uncultured fungus	Basal clone group I	*Chytridiomycota*	*Physoderma dulichii*	DQ536472.1	0.0	91
AB468613	Uncultured fungus	Basal clone group I	*Chytridiomycota*	*Physoderma dulichii*	DQ536472.1	0.0	90
AB468614	Uncultured fungus	Basal clone group I	*Chytridiomycota*	*Mortierella wolfii*	AF113425.1	0.0	91
AB468615	Uncultured fungus	Basal clone group I	*Chytridiomycota*	*Physoderma dulichii*	DQ536472.1	0.0	91
AB468616	Uncultured fungus	Basal clone group I	*Chytridiomycota*	*Physoderma dulichii*	DQ536472.1	0.0	91
AB468617	Uncultured fungus	Basal clone group I	*Chytridiomycota*	*Physoderma dulichii*	DQ536472.1	0.0	91
AB468618	Uncultured fungus	Basal clone group I	*Chytridiomycota*	*Physoderma dulichii*	DQ536472.1	0.0	91
AB468619	Uncultured fungus	Basal clone group I	*Chytridiomycota*	*Physoderma dulichii*	DQ536472.1	0.0	91
AB468620	Uncultured fungus	Basal clone group I	*Chytridiomycota*	*Physoderma dulichii*	DQ536472.1	0.0	90
AB468621	Uncultured fungus	Basal clone group I	*Chytridiomycota*	*Physoderma dulichii*	DQ536472.1	0.0	91
AB468622	Uncultured fungus	Basal clone group I	*Chytridiomycota*	*Physoderma dulichii*	DQ536472.1	0.0	91
AB468623	Uncultured fungus	Basal clone group I	*Chytridiomycota*	*Physoderma dulichii*	DQ536472.1	0.0	91
AB468624	Uncultured fungus	Basal clone group I	*Chytridiomycota*	*Physoderma dulichii*	DQ536472.1	0.0	91
AB468625	Uncultured fungus	Basal clone group I	*Chytridiomycota*	*Physoderma dulichii*	DQ536472.1	0.0	91
AB468626	Uncultured fungus	Basal clone group I	*Chytridiomycota*	*Physoderma dulichii*	DQ536472.1	0.0	91
AB468627	Uncultured fungus	Basal clone group I	*Chytridiomycota*	*Physoderma dulichii*	DQ536472.1	0.0	91
AB468628	Uncultured fungus	Basal clone group I	*Chytridiomycota*	*Rhizophlyctis rosea*	NG_017175.1	0.0	91

AB468629	Uncultured fungus	Basal clone group I	*Chytridiomycota*	*Rhizophlyctis rosea*	NG_017175.1	0.0	91
AB468635	Uncultured fungus	Basal clone group I	*Chytridiomycota*	*Physoderma dulichii*	DQ536472.1	0.0	91
AB468636	Uncultured fungus	Basal clone group I	*Chytridiomycota*	*Physoderma dulichii*	DQ536472.1	0.0	91
AB468637	Uncultured fungus	Basal clone group I	*Chytridiomycota*	*Physoderma dulichii*	DQ536472.1	0.0	91
AB468638	Uncultured fungus	Basal clone group I	*Chytridiomycota*	*Physoderma dulichii*	DQ536472.1	0.0	91
AB468639	Uncultured fungus	Basal clone group I	*Chytridiomycota*	*Physoderma dulichii*	DQ536472.1	0.0	90
AB468640	Uncultured fungus	Basal clone group I	*Chytridiomycota*	*Physoderma dulichii*	DQ536472.1	0.0	91
AB468641	Uncultured fungus	Basal clone group I	*Chytridiomycota*	*Physoderma dulichii*	DQ536472.1	0.0	91
AB468642	Uncultured fungus	Basal clone group I	*Chytridiomycota*	*Physoderma dulichii*	DQ536472.1	0.0	90
AB468643	Uncultured fungus	Basal clone group I	*Chytridiomycota*	*Physoderma dulichii*	DQ536472.1	0.0	90
AB468644	Uncultured fungus	Basal clone group I	*Chytridiomycota*	*Physoderma dulichii*	DQ536472.1	0.0	91
AB468645	Uncultured fungus	Basal clone group I	*Chytridiomycota*	*Physoderma dulichii*	DQ536472.1	0.0	91
AB468646	Uncultured fungus	Basal clone group I	*Chytridiomycota*	*Physoderma dulichii*	DQ536472.1	0.0	90
AB468647	Uncultured fungus	Basal clone group I	*Chytridiomycota*	*Physoderma dulichii*	DQ536472.1	0.0	91
AB468648	Uncultured fungus	Basal clone group I	*Chytridiomycota*	*Physoderma dulichii*	DQ536472.1	0.0	91
AB468649	Uncultured fungus	Basal clone group I	*Chytridiomycota*	*Physoderma dulichii*	DQ536472.1	0.0	91
AB468650	Uncultured fungus	Basal clone group I	*Chytridiomycota*	*Physoderma dulichii*	DQ536472.1	0.0	91
AB468651	Uncultured fungus	Basal clone group I	*Chytridiomycota*	*Physoderma dulichii*	DQ536472.1	0.0	91
AB468652	Uncultured fungus	Basal clone group I	*Chytridiomycota*	*Physoderma dulichii*	DQ536472.1	0.0	91
AB468653	Uncultured fungus	Basal clone group I	*Chytridiomycota*	*Physoderma dulichii*	DQ536472.1	0.0	91
AB468654	Uncultured fungus	Basal clone group I	*Chytridiomycota*	*Physoderma dulichii*	DQ536472.1	0.0	91
AB468655	Uncultured fungus	Basal clone group I	*Chytridiomycota*	*Physoderma dulichii*	DQ536472.1	0.0	91
AB468656	Uncultured fungus	Basal clone group I	*Chytridiomycota*	*Physoderma dulichii*	DQ536472.1	0.0	91
AB468657	Uncultured fungus	Basal clone group I	*Chytridiomycota*	*Physoderma dulichii*	DQ536472.1	0.0	91
AB468658	Uncultured fungus	Basal clone group I	*Chytridiomycota*	*Physoderma dulichii*	DQ536472.1	0.0	91
AB468659	Uncultured fungus	Basal clone group I	*Chytridiomycota*	*Physoderma dulichii*	DQ536472.1	0.0	90
AB468660	Uncultured fungus	Basal clone group I	*Chytridiomycota*	*Physoderma dulichii*	DQ536472.1	0.0	91
AB468661	Uncultured fungus	Basal clone group I	*Chytridiomycota*	*Physoderma dulichii*	DQ536472.1	0.0	91
AB468662	Uncultured fungus	Basal clone group I	*Chytridiomycota*	*Physoderma dulichii*	DQ536472.1	0.0	91
AB468663	Uncultured fungus	Basal clone group I	*Chytridiomycota*	*Physoderma dulichii*	DQ536472.1	0.0	91
AB468664	Uncultured fungus	Basal clone group I	*Chytridiomycota*	*Physoderma dulichii*	DQ536472.1	0.0	90
AB468665	Uncultured fungus	Basal clone group I	*Chytridiomycota*	*Physoderma dulichii*	DQ536472.1	0.0	91
AB468666	Uncultured fungus	Basal clone group I	*Chytridiomycota*	*Physoderma dulichii*	DQ536472.1	0.0	91
AB468667	Uncultured fungus	Basal clone group I	*Chytridiomycota*	*Physoderma dulichii*	DQ536472.1	0.0	91
AB468668	Uncultured fungus	Basal clone group I	*Chytridiomycota*	*Physoderma dulichii*	DQ536472.1	0.0	91
AB468669	Uncultured fungus	Basal clone group I	*Chytridiomycota*	*Physoderma dulichii*	DQ536472.1	0.0	91
AB468670	Uncultured fungus	Basal clone group I	*Chytridiomycota*	*Physoderma dulichii*	DQ536472.1	0.0	91
AB468671	Uncultured fungus	Basal clone group I	*Chytridiomycota*	*Rhizophlyctis rosea*	NG_017175.1	0.0	91
AB468672	Uncultured fungus	*Chytridiomycota*	*Chytridiomycota*	*Rhizophlyctis harderi*	AF164272.2	0.0	92
AB468673	Uncultured fungus	*Chytridiomycota*	*Chytridiomycota*	*Rhizophlyctis harderi*	AF164272.2	0.0	92
AB695460	Uncultured eukaryote	*Cryptomycota*	LKM11	Fungal sp.	JQ796369.1	0.0	91
AB695461	Uncultured eukaryote	LKM15	LKM15	*Hyaloraphidium curvatum*	NG_017172.1	0.0	84
AB695462	Uncultured eukaryote	LKM15	LKM15	*Lamproderma sp.*	AB779691.1	0.0	89
AB695463	Uncultured eukaryote	*Cryptomycota*	LKM11	*Amphora commutata*	AB754836.1	0.0	83
AB695464	Uncultured eukaryote	LKM15	LKM15	*Basidioascus undulatus*	JX242890.1	0.0	85
AB695465	Uncultured eukaryote	*Cryptomycota*	LKM11	Fungal sp.	JQ796368.1	0.0	87
AB695466	Uncultured eukaryote	*Cryptomycota*	LKM11	*Hyaloraphidium curvatum*	NG_017172.1	0.0	89
AB695512	Uncultured eukaryote	*Cryptomycota*	LKM11	Fungal sp.	JQ796369.1	0.0	92
AB695514	Uncultured eukaryote	LKM15	LKM15	*Saitoella complicata*	JQ698935.1	0.0	87
AB695515	Uncultured eukaryote	*Cryptomycota*	LKM11	Fungal sp.	JQ796369.1	0.0	92
AF372712	Uncultured fungus	*Cryptomycota*	LKM11	Fungal sp.	JQ796369.1	0.0	88
AF372713	Uncultured fungus	*Cryptomycota*	LKM11	Fungal sp.	JQ796369.1	0.0	89

AF372716	Uncultured fungus	*Cryptomycota*	LKM11	Fungal sp.	JQ796369.1	0.0	88
AF372717	Uncultured fungus	LKM15	LKM15	*Lamproderma sp.*	AB779691.1	0.0	94
AF372718	Uncultured fungus	LKM15	LKM15	*Lamproderma sp.*	AB779691.1	0.0	94
AJ130849	Uncultured eukaryote	*Cryptomycota*	LKM11	*Hyaloraphidium curvatum*	NG_017172.1	0.0	89
AJ130850	Uncultured eukaryote	LKM15	LKM15	*Lamproderma sp.*	AB779691.1	0.0	99
AJ130857	Uncultured eukaryote	*Cryptomycota*	LKM11	*Saturnispora mendoncae*	EF550352.1	0.0	81
AY180024	Uncultured fungus	*Chytridiomycota*	*Chytridiomycota*	*Entophlyctis confervae-glomeratae*	EF014367.1	0.0	90
AY230211	Uncultured fungus	*Blastocladiomycota*	*Blastocladiomycota*	*Zygomycete sp.*	EU428774.1	0.0	89
AY642700	Unculturedeukaryotic Picoplankton	*Cryptomycota*	LKM11	*Hyaloraphidium curvatum*	NG_017172.1	0.0	89
AY642711	Uncultured eukaryotic Picoplankton	*Cryptomycota*	LKM11	*Saturnispora mendoncae*	EF550352.1	0.0	82
AY642721	Uncultured eukaryotic Picoplankton	*Cryptomycota*	LKM11	Fungal sp.	JQ796369.1	0.0	85
AY642725	Unculturedeukaryotic Picoplankton	*Cryptomycota*	*Chytridiomycota_Incertae Sedis_Rozella*	*Saturnispora mendoncae*	EF550352.1	0.0	80
AY642731	Uncultured eukaryotic Picoplankton	*Cryptomycota*	LKM11	*Candida sp.*	GQ176145.1	0.0	82
AY821991	Uncultured fungus	*Cryptomycota*	LKM11	Fungal sp.	JQ796369.1	0.0	86
AY821992	Uncultured Chytridiomycota	*Blastocladiomycota*	*Blastocladiomycota*	*Blastocadia ramosa*	HQ888695.1	0.0	97
AY821995	Uncultured Chytridiomycota	*Blastocladiomycota*	*Blastocladiomycota*	*Blastocladia pringsheimii*	HQ888696.1	0.0	98
AY821997	Uncultured fungus	*Cryptomycota*	LKM11	*Phaeophleospora eugeniicola*	GU214567.1	0.0	89
DQ244003	Uncultured fungus	*Cryptomycota*	Chytridiomycota_Incertae Sedis_Rozella	*Rhizophydium haynaldii*	KF160859.1	0.0	89
DQ244010	Uncultured fungus	*Blastocladiomycota*	*Blastocladiomycota*	*Porostereum crassum*	AB809163.1	0.0	88
DQ244011	Uncultured fungus	Fungi	Nucletmycea_Fonticula	*Basidiobolus haptosporus*	AF368504.1	0.0	89
DQ244015	Uncultured fungus	*Blastocladiomycota*	*Blastocladiomycota*	*Allomyces macrogynus*	U23936.1	0.0	91
DQ244016	Uncultured fungus	*Cryptomycota*	LKM11	*Candida lusitaniae*	M55526.1	0.0	86
DQ244017	Uncultured fungus	*Cryptomycota*	LKM11	Fungal sp.	JQ796369.1	0.0	88
DQ244018	Uncultured fungus	*Cryptomycota*	LKM11	*Candida incommunis*	AB018133.2	0.0	84
EF100212	Uncultured eukaryote	*Blastocladiomycota*	*Blastocladiomycota*	*Mucoromycotina sp.*	JF414208.1	0.0	92
EF100353	Uncultured eukaryote	LKM15	LKM15	*Lamproderma sp.*	AB779691.1	0.0	87
EF100356	Uncultured eukaryote	*Blastocladiomycota*	*Blastocladiomycota*	*Mortierella alpina*	AJ271630.1	0.0	88
EU050974	Uncultured eukaryote	*Chytridiomycota*	*Chytridiomycota*	*Triparticalcar arcticum*	DQ536480.1	0.0	89
EU091829	Uncultured ban. Eukaryote	*Cryptomycota*	LKM11	*Spizellomyces sp.*	AB586075.1	0.0	90
EU091830	Uncultured ban. Eukaryote	*Chytridiomycota*	*Chytridiomycota*	*Rhizophlyctis rosea*	NG_017175.1	0.0	96
EU091833	Uncultured Banisveld Eukaryote	*Chytridiomycota*	*Chytridiomycota*	*Rhizophlyctis rosea*	NG_017175.1	0.0	96
EU091835	Uncultured Banisveld Eukaryote	*Cryptomycota*	LKM11	Fungal sp.	JQ796369.1	0.0	87
EU091838	Uncultured Banisveld Eukaryote	*Cryptomycota*	LKM11	Fungal sp.	JQ796369.1	0.0	87
EU091850	Uncultured Banisveld Eukaryote	*Cryptomycota*	LKM11	*Candida incommunis*	AB018133.2	0.0	85
EU091851	Uncultured Banisveld Eukaryote	*Cryptomycota*	LKM11	*Spizellomyces sp.*	AB586075.1	0.0	90
EU091852	Uncultured Banisveld Eukaryote	*Cryptomycota*	LKM11	*Spizellomyces sp.*	AB586075.1	0.0	88
EU091865	Uncultured Banisveld Eukaryote	*Cryptomycota*	LKM11	Fungal sp.	JQ796369.1	0.0	93
EU091872	Uncultured Banisveld Eukaryote	*Cryptomycota*	LKM11	Fungal sp,	JQ796369.1	0.0	86
EU154992	Marine Uncultured Fungus	*Blatocladiomycota*	*Chytridiomycota*	*Physoderma dulichii*	DQ536472.1	0.0	91
EU162634	Uncultured fungus	*Cryptomycota*	LKM11	*Hyaloraphidium curvatum*	NG_017172.1	0.0	88
EU162635	Uncultured fungus	*Cryptomycota*	LKM11	*Basidiobolus microsporus*	AF368505.1	0.0	87
EU162639	Uncultured Chytridiomycota	Basal_fungi	Basal_fungi	*Blastocladiales sp.*	EF565163.1	0.0	85
EU910604	Uncultured alveolate	LKM15	LKM15	*Lamproderma sp.*	AB779691.1	0.0	96
EU910606	Uncultured alveolate	LKM15	LKM15	*Lamproderma sp.*	AB779691.1	0.0	99
FJ157332	Uncultured eukaryote	*Chytridiomycota*	*Chytridiomycota*	*Spizellomyces sp.*	AB586075.1	0.0	92
FJ157335	Uncullured eukaryote	*Blastocladiomycota*	*Blastocladiomycota*	*Triparticalcar arcticum*	DQ536480.1	0.0	89
FJ687268	Uncultured fungus	*Cryptomycota*	LKM11	Fungal sp.	JQ796369.1	0.0	90
FJ810604	Uncultured eukaryote	*Cryptomycota*	LKM11	Fungal sp.	JQ796369.1	0.0	93
FJ810611	Uncultured eukaryote	*Mucoromycotina*	*Mucoromycotina*	*Mortierella sp.*	AB521052.1	0.0	99
FJ810613	Uncultured eukaryote	*Cryptomycota*	LKM11	Fungal sp.	JQ796369.1	0.0	89
FJ810615	Uncultured eukaryote	*Cryptomycota*	LKM11	Fungal sp.	JQ796369.1	0.0	90
FJ976648	Uncultured fungus	*Cryptomycota*	Chytridiomycota_Incertae Sedis_Rozella	*Rhizophlyctis rosea*	NG_017175.1	0.0	91

FJ976649	Uncultured opisthokont	*Cryptomycota*	LKM11	*Rhizophydium sphaerotheca*	AY635823.1	0.0	88
FJ976650	Uncultured opisthokont	*Blastocladiomycota*	*Blastocladiomycota*	*Spizellomyces sp.*	AB586075.1	0.0	89
FR874399	Uncultured marine Picoeukaryote	*Cryptomycota*	LKM11	Fungal sp.	JQ796369.1	0.0	93
FR874575	Uncultured marine Picoeukaryote	*Cryptomycota*	LKM11	Fungal sp.	JQ796369.1	0.0	93
FR874713	Uncultured marine Picoeukaryote	*Cryptomycota*	LKM11	Fungal sp.	JQ796369.1	0.0	93
FR874714	Uncultured marine Picoeukaryote	*Cryptomycota*	LKM11	*Fungal sp.*	JQ796369.1	0.0	93
GQ330607	Uncultured nucleariidae	*Nuclearia*	Nucletmycea_Nuclearia	*Nuclearia sp.*	HG530253.1	0.0	97
GQ330608	Uncultured nucleariidae	*Nuclearia*	Nucletmycea_Nuclearia	*Nuclearia simplex*	AF484687.1	0.0	92
GQ330622	Uncultured cf. *Mortierella*	*Mucoromycotina*	*Mucoromycotina*	*Mortierella parvispora*	AY129549.1	0.0	99
GQ330623	Uncultured cf. *Umbelllopsis*	*Mucoromycotina*	*Mucoromycotina*	*Umbelopsis ramanniana*	NG_017189.1	0.0	99
GQ330624	Uncultured cf. *Umbelllopsis*	Basal_fungi	Basal_fungi	*Basidiobolus magnus*	JX242608.1	0.0	88
GQ330641	Uncultured eukaryote	Eukaryote	Nucletmycea_Fonticula	*Cryptosporidium sp.*	AB222185.1	0.0	85
GU067817	Uncultured fungus	*Blastocladiomycota*	*Blastocladiomycota*	*Triparticalcar arcticum*	DQ536480.1	0.0	89
GU067910	Uncultured fungus	*Blastocladiomycota*	*Blastocladiomycota*	*Basidiobolus microsporus*	AF368505.1	0.0	91
GU067912	Uncultured fungus	*Blastocladiomycota*	*Blastocladiomycota*	*Flammulina velutipes*	HQ660197.1	0.0	84
GU067917	Uncultured fungus	*Blastocladiomycota*	*Blastocladiomycota*	*Hyaloraphidium curvatum*	NG_017172.1	0.0	89
GU067928	Uncultured fungus	*Blastocladiomycota*	*Blastocladiomycota*	*Triparticalcar arcticum*	DQ536480.1	0.0	92
GU067958	Uncultured fungus	*Blastocladiomycota*	*Blastocladiomycota*	*Basidiobolus microsporus*	AF368505.1	0.0	91
GU067976	Uncultured eukaryote	*Blastocladiomycota*	*Blastocladiomycota*	*Coriolopsis gallica*	AY336772.1	0.0	85
GU067982	Uncultured fungus	*Cryptomycota*	Chytridiomycota_Incertae Sedis_Rozella	*Rhizophydium elyensis*	DQ536479.1	0.0	90
HM443426	Uncultured fungus	*Chytridiomycota*	*Chytridiomycota*	*Basidiobolus haptosporus*	AF113413.1	0.0	90
HM486975	Uncultured fungus	*Chytridiomycota*	*Chytridiomycota*	*Olpidium brassicae*	NG_017176.1	0.0	99
HM486987	Uncultured fungus	LKM15	LKM15	*Lamproderma sp.*	AB779691.1	0.0	94
HM486989	Uncultured fungus	*Cryptomycota*	LKM11	Fungal sp.	JQ796369.1	0.0	90
HM486990	Uncultured fungus	*Cryptomycota*	LKM11	*Lamproderma sp.*	AB779691.1	0.0	92
HM486991	Uncultured fungus	*Cryptomycota*	LKM11	Fungal sp.	JQ796369.1	0.0	90
HM486992	Uncultured fungus	*Cryptomycota*	LKM11	*Endogone aggregata*	JF414195.1	0.0	90
HM486993	Uncultured fungus	*Cryptomycota*	LKM11	*Endogone aggregata*	JF414195.1	0.0	90
HM486994	Uncultured fungus	*Cryptomycota*	LKM11	Fungal sp.	JQ796369.1	0.0	90
HM486995	Uncultured fungus	*Cryptomycota*	LKM11	*Endogone aggregata*	JF414195.1	0.0	90
HM487005	Uncultured fungus	*Chytridiomycota*	*Chytridiomycota*	*Rhizophlyctis rosea*	NG_017175.1	0.0	93
HM487006	Uncultured fungus	LKM15	LKM15	*Lamproderma sp.*	AB779691.1	0-0	97
HM487007	Uncultured fungus	LKM15	LKM15	*Lamproderma sp.*	AB779691.1	0.0	99
HM487008	Uncultured fungus	LKM15	LKM15	*Lamproderma sp.*	AB779691.1	0.0	98
HM487009	Uncultured fungus	LKM15	LKM15	*Lamproderma sp.*	AB779691.1	0.0	97
HM487010	Uncultured fungus	LKM15	LKM15	*Lamproderma sp.*	AB779691.1	0.0	95
HM487011	Uncultured fungus	LKM15	LKM15	*Lamproderma sp.*	AB779691.1	0.0	99
HM487046	Uncultured fungus	LKM15	LKM15	*Lamproderma sp.*	AB779691.1	0.0	97
HM487047	Uncultured fungus	LKM15	LKM15	*Lamproderma sp.*	AB779691.1	0.0	97
HM487048	Uncultured fungus	*Cryptomycota*	LKM11	*Endogone aggregata*	JF414195.1	0.0	90
HM487049	Uncultured fungus	*Cryptomycota*	LKM11	Fungal sp.	JQ796369.1	0.0	90
HM487051	Uncultured fungus	*Cryptomycota*	LKM11	*Spizellomycete sp.*	DQ536490.1	0.0	90
HM487053	Uncultured fungus	LKM15	LKM15	*Lamproderma sp.*	AB779691.1	0.0	99
HQ191302	Uncultured eukaryote	*Blastocladiomycota*	*Blastocladiomycota*	*Catenomyces sp.*	AY635830.1	0.0	91
JN090865	Uncultured eukaryote	*Blastocladiomycota*	*Blastocladiomycota*	*Triparticalcar arcticum*	DQ536480.1	0.0	92
JN090896	Uncultured eukaryote	Basal_fungi	Basal_fungi	*Basidiobolus haptosporus*	AF368504.1	0.0	90
JN090898	Unculltured eukaryote	*Blastocladiomycota*	*Blastocladiomycota*	*Triparticalcar arcticum*	DQ536480.1	0.0	90
JN705518	Uncultured Microeukaryote	*Entomophthoromycotina*	*Entomophthoromycotina*	*Conidiobolus firmipilleus*	AF368507.1	0.0	99
JN705541	Uncultured Microeukaryote	LKM15	LKM15	*Lamproderma sp.*	AB779691.1	0.0	87
